# Dietary fiber for the prevention of childhood obesity: a focus on the involvement of the gut microbiota

**DOI:** 10.1080/19490976.2024.2387796

**Published:** 2024-08-20

**Authors:** Zhongmin Yang, Mingyue Yang, Edward C. Deehan, Chenxi Cai, Karen L. Madsen, Eytan Wine, Guiling Li, Jian Li, Jingwen Liu, Zhengxiao Zhang

**Affiliations:** a College of Ocean Food and Biological Engineering, Jimei University, Xiamen, Fujian, China; b Department of Food Science and Technology, University of Nebraska, Lincoln, NE, USA; c Nebraska Food for Health Center, University of Nebraska, Lincoln, NE, USA; d School of Public Health, Xiamen University, Xiamen, Fujian, China; e Division of Gastroenterology, Department of Medicine, University of Alberta, Edmonton, AB, Canada; f Division of Pediatric Gastroenterology, Departments of Pediatrics and Physiology, University of Alberta, Edmonton, AB, Canada; g Fujian Provincial Engineering Technology Research Center of Marine Functional Food, Xiamen, Fujian, China

**Keywords:** Dietary fiber, gut microbiota, childhood, obesity, precision nutrition

## Abstract

Given the worldwide epidemic of overweight and obesity among children, evidence-based dietary recommendations are fundamentally important for obesity prevention. Although the significance of the human gut microbiome in shaping the physiological effects of diet and obesity has been widely recognized, nutritional therapeutics for the mitigation of pediatric obesity globally are only just starting to leverage advancements in the nutritional microbiology field. In this review, we extracted data from PubMed, EMBASE, Scopus, Web of Science, Google Scholar, CNKI, Cochrane Library and Wiley online library that focuses on the characterization of gut microbiota (including bacteria, fungi, viruses, and archaea) in children with obesity. We further review host-microbe interactions as mechanisms mediating the physiological effects of dietary fibers and how fibers alter the gut microbiota in children with obesity. Contemporary nutritional recommendations for the prevention of pediatric obesity are also discussed from a gut microbiological perspective. Finally, we propose an experimental framework for integrating gut microbiota into nutritional interventions for children with obesity and provide recommendations for the design of future studies on precision nutrition for pediatric obesity.

## 1. Introduction

The presence of childhood obesity is a prominent risk factor for the development of obesity-related comorbidities in adolescence, including increased risk of type 2 diabetes^1^, nonalcoholic fatty liver disease,^2^ and cardiovascular disease.^3^ Data released by the World Obesity Federation reported that the increase in the prevalence of obesity is expected to be steepest among children and adolescents aged 5–19 years, rising from 10% to 20% of the world’s boys and from 8% to 18% of the world’s girls between 2020 and 2035.^4^ Recent research further suggests that having excess body weight in childhood and adolescence was not only found to adversely affect physical health later in life^5^ but also negatively impact psychosocial wellbeing.^6^ Nevertheless, current strategies for treating childhood obesity, such as lifestyle changes and pharmacotherapy, have very modest effectiveness,^7^ concerning side effects,^8^ or inconsistent outcomes.^9^ Identifying the causes of childhood obesity and targeting interventions is, therefore, important in the context of preventive health.

Various factors have been suggested as potential causes of obesity, including lifestyle choices and genetic factors,^10^ with the gut microbiota emerging as an important factor contributing to the pathogenesis of obesity.^11^ Due to their profound effects on the host, this highly complex group of microbes that reside along the human gastrointestinal tract is referred to as an individual’s “second genome”.^12^ The gut microbiota is a community of more than 10^13^ microorganisms that includes bacteria, fungi, viruses, and archaea, which live as a complex ecosystem with the human host.^6,13^ There is accumulating evidence that the gut microbiota affects all aspects of energy homeostasis through mechanisms involving immune, hormonal and neural systems, and that gut dysbiosis, or an abnormal composition of gut microbial taxa might contribute to a disturbed host metabolism through effects on adipose tissue, muscle, and liver.^14^

Although growing evidence from animal models that the gut microbiota has a potential causal role in obesity and would be a promising target for precision therapeutics, there is still a lack of convincing evidence for a direct contribution of the gut microbiota to humans with obesity.^15^ Whether gut microbial alteration exists in children with obesity is yet to be systematically evaluated.^16,17^ Moreover, the gut microbiome is thought to stabilize and resemble that of an adult after the first 1 to 3 years of life,^18^ while other research suggests that it continues to develop throughout adolescence and that gaining excess body weight during childhood is likely to play a role in further shaping the gut microbiota.^19,20^ It is unclear what role the coexistence of gut cross-kingdom community interactions in the gut plays in childhood obesity. Therefore, identifying the gut microbiota characteristics that are conserved in children with obesity and also play a pathogenic role in the development of childhood obesity is a challenge in the field. This has important implications for the development of microbiome-targeted therapies for the prevention and treatment of childhood obesity.

Dietary substances, such as fibers and prebiotics, have been found to induce a positive impact on the children gut microbiota.^21^ As a result, they hold potential for the management of obesity. Dietary fibers (DFs), carbohydrate polymers that are neither digested nor absorbed in the small intestine, are subject to microbial fermentation in the human colon and, thus, influence the composition of microbial communities as well as their metabolic activities.^22^ Some DFs can also be classified as prebiotics,^23^ or substrates selectively used by host microorganisms that confer a health benefit to humans.^23^ However, currently it is not clear if the beneficial effects of increasing DF intake are due to microbial metabolism and the production of metabolites such as short-chain fatty acids (SCFAs) or due to a DF-altered intestinal transit time, changes in nutrient absorption, fecal bulking and binding of various metabolites, or modulation of the immune system.^18,24^

It is crucial to keep in mind that the physiological functions of DFs are highly structure-dependent. Subtle variations in the chemical structures of DFs affect their utilization by gut microbes, as bacteria have different abilities to cleave the linkages in the structure of these complex molecules to obtain simple sugars.^25^ Simultaneously, recent reviews have revealed that intrinsic and isolated fibers have functional specificity as well.^26^ However, there is insufficient literature evidence to support the targeted modulatory effects of DFs on the gut microbiota of children with obesity. Furthermore, adults are encouraged to consume at least 25–38 g of DFs per day, but there is currently a lack of data to support DF guidelines for children in most countries. As a result, more research is needed to thoroughly investigate the relationship between DFs and childhood obesity from a gut microbiota perspective.

In studies of children with obesity, examination of DF-microbiota-host interactions has been limited, despite recent increases in nutritional microbiology research in adults. In this perspective, we first systematically evaluated whether special microbial alterations exist in children with obesity, and then reviewed the effects of DFs and DFs-related patterns on the gut microbiota in children with obesity. We further apply this information to confront an ongoing debate in the field of childhood obesity research. Finally, we propose a microbiome-targeted research approach for evaluating treatments for the reduction of childhood obesity and outline an experimental framework for systematically incorporating the gut microbiome into future nutrition research.

## 2. Systematic review: characterization of the gut microbiota in children with obesity

We conducted a systematic literature review in July 2022 (with an updated literature search in June 2024) to assess the pertinent gut microbiota traits of childhood obesity (see Box 1 for search strategy). Of the 63 studies that met the inclusion criteria, some of the gut microbes in children with obesity showed consistent trends (e.g., Firmicutes/Bacteroidetes (F/B) ratios, *Akkermansia muciniphila*, *Lactobacillus* spp., *Dialister*, etc). However, there is considerable heterogeneity among the findings of most published studies investigating gut microbiota in childhood obesity. Understanding the compositional characteristics of the gut microbiota has the potential to help determine their role in the onset or development of obesity in the host, laying the groundwork for microbiome-based therapies for childhood obesity to improve the quality of life.

**Table:** 

Box 1 To identify gut microbiota signatures that are characteristics of childhood obesity, we conducted a systematic review. We included all retrieved studies that reported on the gut microbiota of children with obesity, which included cross-sectional and case-control studies. In total, 63 studies (all were observational studies) that assessed changes in the gut microbiome of children with obesity were included in this review for analysis: 67% of studies (*n* = 42) used 16S rRNA sequencing for microbial analysis,^27–68^ nine studies used Real-time PCR (RT-PCR),^69–77^ six studies used shotgun metagenomic sequencing,^78–83^ and the remaining six studies used other methods to assess microbial differences.^84–89^ Information on each study in this review is included in **Table S1** in the supplementary materials. **Box 1 Figure**. Flow Diagram of Article Search and Selection Process. 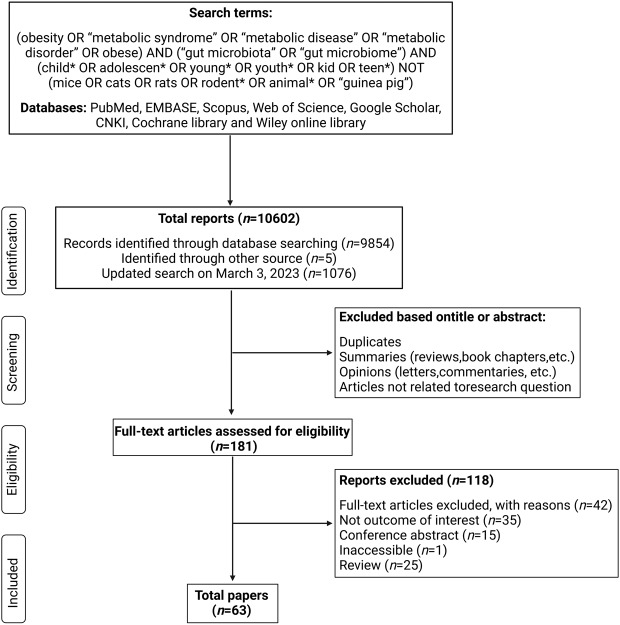 The literature search was conducted in July 2022, and an updated literature search was conducted in June 2024.

Summarized in Figure S1 are the main findings of the included studies regarding the relationship between microorganisms and childhood obesity. Only microbial features reported to correlate with obesity in three or more of the publications reviewed were included. The figure summarizes the percentage of included studies reporting the gut microbiota α-diversity, bacteria (7 phyla, 30 genera, 12 species), and archaea (1 species) in children with obesity in agreement with the indicated relationship with adiposity. The proceeding detailed analysis is grouped by gut bacterial diversity and compositional characteristics of bacteria, fungi, viruses, and archaea characteristics in children with obesity.

### 2.1. Microbial diversity

**Figure 1.**
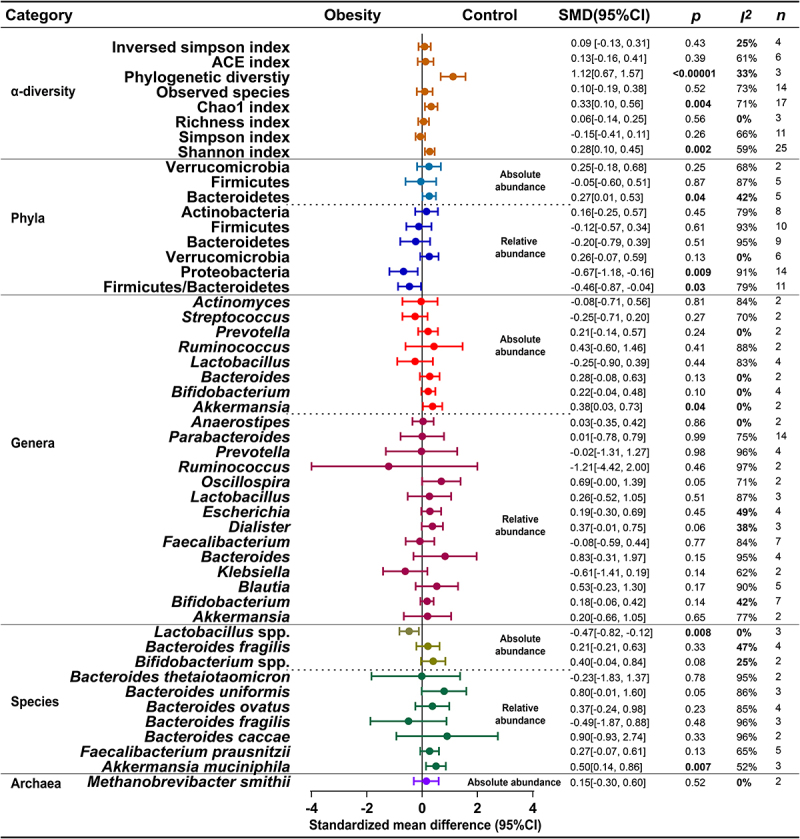
Meta-analysis of gut microbiota differences and α-diversity between obese children and controls. SMD, standardized mean difference. CI, confidence interval. The “*p*” indicates the level of significance. The “*I*^*2*^” indicates the results of the heterogeneity test. The “*n*” indicates the number of studies that provided usable data.

Of the 63 studies included, 65% (41/63) reported α-diversity in children with and without obesity (Table S1). Eight indices were used to assess α-diversity, and the most widely applied were Chao1, Shannon, Simpson and Observed species (Figure S1). There was no evidence of publication bias in any of the analyses (Figure S2). We conducted a meta-analysis based on the available α-diversity index data (Figure 1 and Figure S3). The results revealed a statistically significant difference in Phylogenetic diversity, Chao1 index, and Shannon index of gut microbiota α-diversity index between children with obesity and control individuals. Compared to control, Phylogenetic diversity (6 studies with 3 estimates provided data, SMD = 1.12, 95% CI 0.67, 1.57, *I*^*2*^ = 33%, *p* < 0.00001), Shannon index (32 studies with 25 estimates provided data, SMD = 0.28, 95% CI 0.10, 0.45, *I*^*2*^ = 59%, *p* = 0.002), Chao1 index (22 studies with 17 estimates provided data, SMD = 0.33, 95% CI 0.10, 0.56, *I*^*2*^ = 71%, *p* = 0.004) and were significantly decreased in children with obesity. Considering the heterogeneity, sensitivity analyses were performed by omitting each study in turn, which showed stability for the Chao1 index, and a decrease in heterogeneity for the Shannon index by omitting Méndez-Salazar 2018^43^ (*p* = 0.002, *I*^*2*^ = 42%) (Figure S4). Based on the results of regional subgroup analyses, there were statistically significant differences for the Shannon index in Asian (*p* = 0.0002, *I*^*2*^ = 41%) and European (*p* = 0.01, *I*^*2*^ = 0%), and Simpson index (*p* = 0.001, *I*^*2*^ = 15%) in North America, with low heterogeneity. No significant differences or high heterogeneity were observed in any of the results based on age and BMI subgroup analyses (Figure S5).

Strong microbiota diversity is critical to the ability of the gut microbiota to adapt to stress and is a key indicator of good health. Meta-analysis showed a significant decrease in the Shannon index, Chao1 index and Phylogenetic diversity in obese children, aligning with the prevailing hypothesis. It is important to emphasize that different α-diversity indices exhibit different microbiota characteristics. For example, while the Chao 1 index is based on the total number of bacteria within a community, the Shannon index and the phylogenetic diversity index also consider bacterial evenness and phylogenetic abundance, respectively. Therefore, despite heterogeneity between studies, including differences in region, gender and age, statistically significant differences between alpha diversity indices suggest that ecological disorder in obese children is expressed through disruption of phylogenetic abundance, bacterial homogeneity, and changes in bacterial numbers. However, the common assumption that “more diversity is better” may oversimplify the intricate complex mechanisms.^90^ Measurement of diversity in children with obesity should be used as a starting point for further research of gut micro-ecological mechanisms in obesity and will help to explore causal relationships between the gut microbiome and its host. Furthermore, the variation between the diversity of gut microbiota in obese children and adults deserves further exploration.^91,92^

Beta diversity comparison between childhood obesity and controls was reported in 43% (27/63) studies (Table S1). 17 studies reported consistent significant differences of obese versus non-obese children, while 10 studies reported conflicting results. The results of these studies suggest differences in the common phylogenetic structure of obese children compared to controls. However, the method of measurement, geographic region, age, and degree of obesity may influence the outcomes.

### 2.2. Variations in bacterial composition

Meta-analysis based on relative or absolute abundance data at the phyla level between childhood obesity and controls revealed statistically significant differences for Bacteroidetes, Proteobacteria and F/B ratios (Figure 1 and Figure S6). Compared to control individuals, F/B ratios (23 studies with 11 estimates provided data, SMD = −0.46, 95% CI −0.87, −0.04, *I*^*2*^ = 79%, *p* = 0.03) and relative abundance of Proteobacteria (21 studies with 14 estimates provided data, SMD = −0.67, 95% CI −1.18, −0.16, *I*^*2*^ = 91%, *p* = 0.009) were significantly increased, and absolute abundance of Bacteroidetes (36 studies with 5 estimates provided data, SMD = 0.27, 95% CI 0.01, 0.53, *I*^*2*^ = 42%, *p* = 0.04) was significantly decreased in obese children. Considering the high heterogeneity, sensitivity analyses were performed by omitting each study in turn, with stable results (Figure S7). No publication bias in F/B ratios, relative abundance of Firmicutes and Proteobacteria (Figure S8). Based on the results of region, age and BMI subgroup analyses, there was a significant difference in F/B ratios (*p* < 0.00001, *I*^*2*^ = 0%) in European, and no significant difference between relative abundance of Firmicutes (*p* = 0.89, *I*^*2*^ = 0%) in school-aged children and absolute abundance of Firmicutes (*p* = 0.63, *I*^*2*^ = 0%) in overweight children, with high heterogeneity of the results of the analyses of the other subgroups (Figure S9).

At the genera level, the absolute abundance of *Akkermansia* (6 studies providing 2 estimates, SMD = 0.38, 95% CI 0.03, 0.73, *I*^*2*^ = 0%, *p* = 0.04) was significantly decreased in obese children only compared with controls (Figure 1 and Figure S10). Sensitivity analysis showed a significant increase relative abundance of *Faecalibacterium* by omitting Tian 2023^65^(*p* = 0.0004, *I*^*2*^ = 0%) (Figure S11). Region subgroup analyses showed low heterogeneity and significant increase relative abundance of *Faecalibacterium* in North America (*p* = 0.02, *I*^*2*^ = 21%) (Figure S12).

At the species level, *Akkermansia muciniphila* (4 studies) was consistently decreased in children with obesity (Figure S1). Meta-analysis results showed that relative abundance of *Akkermansia muciniphila* (4 studies providing 3 estimates, SMD = 0.50, 95% CI 0.14, 0.86, *I*^*2*^ = 52%, *p* = 0.007) was significantly decreased and absolute abundance of *Lactobacillus* spp. (3 studies providing 3 estimates, SMD = −0.47, 95% CI −0.82, −0.12, *I*^*2*^ = 0%, *p* = 0.008) was significantly increased in obese children compared to controls (Figure 1 and Figure S13). Sensitivity analyses showed a significant decrease relative abundance of *A. muciniphila* (*p* < 0.0001, *I*^*2*^ = 0%) and *Bacteroides ovatus* (*p* < 0.0001, *I*^*2*^ = 0%) after omitting López-Contreras 2018^45^ (Figure S14). Region subgroup analyses demonstrated low heterogeneity and a significant decrease in the relative abundance of *Faecalibacterium prausnitzii* among Asian individuals (*p* = 0.0002, *I*^*2*^ = 21%) (Figure S15).

Clinical studies have demonstrated notable benefits in overweight/obese individuals supplementing with pasteurizing *A. muciniphila* bacteria to significantly improve insulin resistance, blood cholesterol and inflammatory factor levels.^93^ It is estimated that *F. prausnitzii* makes up 1% to 6% of the total microbiota of healthy individuals,^94^ and has been shown to impart anti-inflammatory characteristics in part by the production of butyrate.^94,95^ However, these anti-inflammatory benefits are not observed in children with obesity. Despite presumably sharing a core microbiome architecture, recent research has indicated that the gut microbiota of children between the ages of 3 and 18 is distinct in terms of both function and taxonomy compared to that of adults.^20^ This suggests that the establishment of the gut microbiota may require a somewhat lengthy period. A meta-analysis of data from adults with obesity revealed that the abundance of *Dore*a, *Dialister*, *Fusobacterium*, *Sutterella*, *Streptococcus*, and *Prevotella* was significantly increased in obesity. Conversely, *Bifidobacterium* exhibited a lower abundance.^92^ In contrast, in children with obesity, *Dialister*, *Streptococcus*, and *Prevotella* did not observe a similar pattern of changes as in adults with obesity (Figure 1), indicating a disparity between the gut microbiota of children and adults with obesity.

### 2.3. Variations in fungi composition

Human gut fungi are receiving increased research attention^96,97^ due to their potential involvement in the etiology of numerous gut-associated diseases.^98^ *Candida* is a diploid fungus that causes disease by invading the host’s mucous membranes and tissues, triggering local and systemic inflammatory responses.^99^ There are many species of fungi in the genera *Candida*, but only a few are pathogenic to humans, mainly *Candida albicans*, *Candida tropicalis*, *Candida parapsilosis*, and *Candida dubliniensis*.^100^ Only 5% (3/63) of the systematically reviewed studies investigated the differences in fungi composition between children with and without obesity. As there were fewer than three studies for the same fungi, these studies were not included in Figure S1. The study by Grigorova *et al*.^51^ reported that *Candida* was not detected in any of the tested feces specimens from children with obesity, while in the control group three *Candida* species were detected: *C. albicans*, *Candida glabrata*, and *Candida krusei*. Borgo *et al*.^88^ also found a relatively low abundance of *C. albicans* and *Saccharomyces cerevisiae* in children with obesity compared to the normoweight children. Furthermore, *C. parapsilosis* and *C. glabrata*were detected in normoweight children but not children with obesity. These findings are contrary to research in mice that show *C. parapsilosis* stimulates the formation of fatty acids by producing a fungal lipase that causes obesity.^101^ Peng *et al*.^38^ report that *Basidiomycota* sp. was more abundant in children with obesity. Reporting gut mycobiota in human fecal specimens and its presence and relative abundance is currently nascent without a consensus on what is considered an “ideal mycobiome”.^102^

Previously, in vitro culturing was the major method used by researchers to study fungi from the intestinal environment. Despite recent breakthroughs in molecular approaches that eliminate the need to cultivate bacteria, this research has mostly concentrated on the non-fungi component of this ecosystem. Consequently, few studies have included the gut mycobiome and even fewer in the pediatric population. It is noteworthy to emphasize that the gut is a diverse and intricate ecosystem within the human body. Research findings indicate that the most prevalent genera in the mycobiome of a healthy adult gut include *Candida*, *Saccharomyces*, and *Cladosporium*.^103^ *Candida* spp. continues to be identified as the prevailing fungi species among individuals aged 65 years and older.^104^ Nevertheless, in diseases such as obesity^105^ and inflammatory bowel disease,^106^ an increase in *Candida* spp. and its inverse correlation with bacterial microbiome diversity have been repeatedly observed. Some studies suggest that *Candida* spp. may break down starch in foods, releasing simple sugars that are fermented by other bacteria.^107^ Furthermore, *C. albicans* was observed to decrease the levels of dissolved oxygen in its immediate environment, creating favorable conditions for the proliferation of anaerobic bacteria, such as *Clostridioides difficile*.^108,109^ Overall, observational and exploratory investigations of gut fungi in children with obesity have highlighted the significant role of dysbiosis of the gut microbes in the pathogenesis of obesity. Understanding cross-kingdom microbial interactions between gut microorganisms in pediatric obesity is important for elucidating the relationship between obesity and microbiota.

### 2.4. Variations in viruses composition

Human feces contain approximately 10^8^ to 10^9^ viruses per gram of feces with DNA bacteriophages predominating and RNA viruses being only a minor fraction of the gut.^110,111^ Maya-Lucas *et al*.^78^ noted that the relative abundance of human herpesvirus 4 (NC-007605.1) increased from 0.08% in normoweight children to 1.53% in children with obesity, while Torque teno midi virus 1 (NC-009225.1) decreased from 2.21% in normoweight children to 0.72% in children with obesity. Furthermore, specific virus particles, notably bacteriophages, might play an essential role in the maintenance of certain strains of gut bacteria associated with the development of obesity.^112^ Shirley *et al*.^113^ used metagenomic sequencing to evaluate virus-like particles (VLPs) in the feces of children with obesity. The results indicate an increase in the prevalence and variety of all phage categories in children with obesity and metabolic syndrome. In the same cohort of children, it was observed that the virome of children with obesity was predominantly dominated by Caudovirales, with only Inoviridae showing a significant increase in the number of viruses.^114^ Intriguingly, further analysis of the enteroviruses of the same cohort of children revealed that loss of CrAssphage stability was associated with obesity, with the effect being more pronounced in cases of obesity accompanied by metabolic syndrome.^115^ However, additional research is required to comprehend the underlying reasons behind the decrease in CrAssphage variety and abundance in individuals with obesity and metabolic syndrome, which contradicts the observed patterns in all phagosomes.

The enteric viruses and phage-bacteria interaction are also crucial for health effects. According to a study by Dutilh et al.,^116^ “CrAss-like” phages can infect Bacteroidetes taxa such as *Prevotella intermedia* and *Bacteroides* spp. and persist inside these hosts. Phages can coexist with gut bacteria for long periods and have the potential to modulate the gut microbiota.^117^ Furthermore, it has been suggested that successful treatments against *Clostridioides difficile* utilizing bacteria-free fecal filtrate imply that phagosome modification may be a useful therapeutic technique to stabilize bacterial eubiotics in the microbiome.^118^ As in other niches, phages play a crucial part in childhood obesity and the disorders that accompany it. Although the nature of all viruses and their role in the health or dysbiosis states of the intestinal microbiome are not currently known, the evidence indicates that their role may be fundamental for bacterial dynamics, pointing to the need to perform deep gut virome studies to elucidate their ecological role in this niche.

### 2.5. Variations in archaea composition

Methanobacteriales are the most abundant order of archaea and their members can produce methane, reduced CO_2_ or methanol with H_2_ as the main electron donor. The main methanogens in the gut microbiota are *Methanobrevibacter smithii*,^119^ *Methanosphaera stadtmanae*,^120^ and *Methanomassilicoccus luminyesis*.^121^ Animal studies have suggested that archaea, specifically *M. smithii*, may play a role in the development of obesity. Samuel and Gordon^122^ observed that* M. smithii *playeda critical role in facilitating an increased capacity of *B. thetaiotaomicron* to digest polyfructose-containing glycans leading to increased production of SCFAs and total liver triglycerides in mice.

However, meta-analysis showed no statistically significant difference in the absolute abundance of *Methanobrevibacter smithii* (3 studies providing 2 estimates, SMD = 0.15, 95% CI −0.30, 0.60, *I*^*2*^ = 0%, *p* = 0.52) between obese children and controls (Figure 1). In addition, there was an increase in the relative abundance of a different unclassified species of *Methanobrevibacter* spp. in children with obesity.^78^ Members of this genera have productive saccharolytic activity, allowing polysaccharide digestion in the gut.^123^ Although the reviewed studies have not yielded similar findings as observed in animals, it should be noted that this does not negate the potential significance of methanogens in metabolic dysregulation among children with obesity. However, it is important to acknowledge that the current data available are limited and further research is warranted to elucidate their precise role in obesity.

### 2.6. Childhood obesity and gut microbiota: outstanding questions

In this review report, although we identified some gut microbiota characteristics of pediatric obesity, it is important to note that our target population is in broad and diversified in terms of physiological developmental stages. The reported results exhibit a high degree of heterogeneity, with confounding factors such as diet, geography, age, sample size, and testing methods often influencing the composition of gut microbiota. Furthermore, whether changes in the gut microbiota precede the development of obesity in children or if they reflect the obese phenotype as a whole remains to be answered. The interrelationship between nutrition, microbiota, genetic factors, immunity, host life behaviors, and the development of childhood obesity is complex, making it challenging to provide definitive conclusions on this subject. Furthermore, the primary succession of the gut microbiota in the human life cycle ends with the formation of the climacteric community, which is thought to be achieved during adolescence and largely maintained into adulthood; this community is characterized by relative stability.^18,124^ Therefore, this remains a very challenging area of research, not only to determine the causal relationship between gut microbiota and obesity but also to propose potential effective interventions that can host a healthier microbiota from childhood and thus promote a better metabolic balance of the organism.

## 3. Effects of dietary fibers on the gut microbiota of children with obesity

### 3.1. Microbial metabolism of dietary fibers and obesity implications

**Figure 2.**
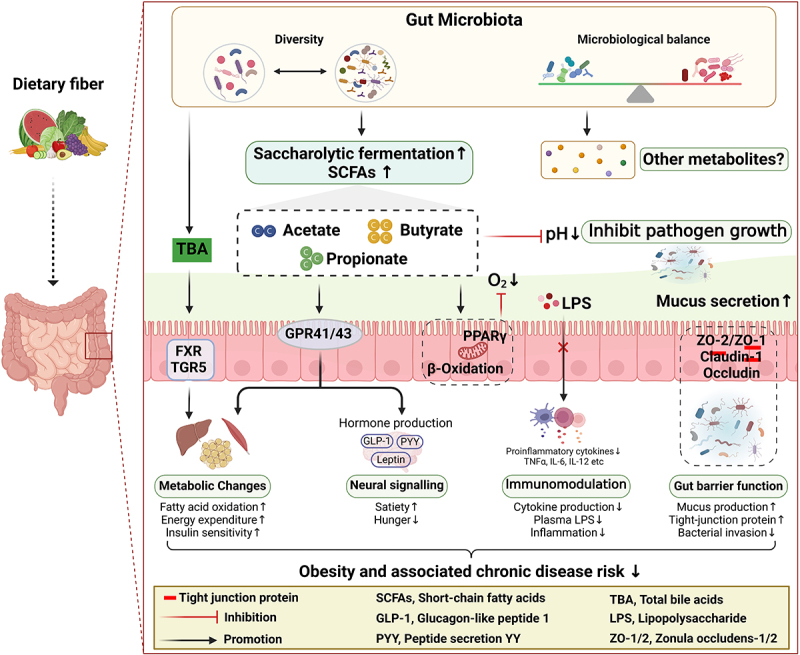
A summary of the mechanisms by which DFs initiate metabolic changes to combat the development of childhood obesity. These mechanisms include the production of microbial metabolic products, noting SCFAs, a decrease in luminal pH and O_2_, regulation of the immune system (modulating cytokine production), improving gut barrier function, promoting satiety through gut-brain signaling, and enhancing oxidative metabolism.

DFs serve as a crucial energy source for microorganisms residing in the gastrointestinal tract. Under specific intestinal conditions, anaerobic bacteria ferment complex carbohydrates by activating mechanisms consisting of critical enzymes and metabolic pathways that produce metabolites such as SCFAs (Figure 2).

SCFAs are organic compounds composed predominantly of acetate, propionate, and butyrate, and one of their mechanisms of action in obesity is to regulate intestinal endocrine signals, influencing glucose and lipid metabolism. Acetate, butyrate, and propionate can interact with G protein-coupled receptors (GPCR41, GPCR43)^125–127^ leading to the activation of anorexigenic hormones such as glucagon-like peptide 1 (GLP-1) and peptide secretion YY (PYY).^128,129^ These hormones play a crucial role in regulating appetite, reducing energy intake and helping to promote glucose metabolism.^130^ SCFAs further improve oxidative metabolism and insulin sensitivity in the liver and adipose tissue by interacting with the G protein-coupled receptor protein FFAR3 and promoting uncoupling protein 2 (UCP2) activity.^131^ Another mechanism of action of SCFA in improving obesity is the reduction of metabolic endotoxemia and inflammation. Obesity is thought to lead to a “leaky gut” and subsequent metabolic endotoxemia (increased serum Lipopolysaccharides [LPS] levels) in animal models and to some extent in humans.^132^ LPS appears to bridge the gap between the gut microbiota and inflammation, and once it is translocated into systemic circulation, it binds to Toll-like receptor 4 and initiates the production of pro-inflammatory cytokines.^133^ SCFAs also play an anti-inflammatory role by regulating the size and function of the colonic regulatory T cells, leading to a decrease in pro-inflammatory factors (e.g., IL-6, IL-8) in intestinal epithelial cells and metabolic tissues (e.g., adipose tissue).^134^ Furthermore, SCFAs play a crucial role in preserving the integrity of the intestinal mucus barrier and creating a favorable milieu that supports the proliferation of putatively beneficial gut microbes. *Bacteroides thetaiotaomicron* produces acetate and propionate, which promote differentiation of goblet cells and stimulate mucin secretion. In contrast, *F. prausnitzii* utilizes acetate to generate butyrate, which attenuates the effect of *B. thetaiotaomicron* on mucus and helps the epithelial cells to maintain the proper ratio of different types of cells in the secretory lineage, thereby reducing mucus overproduction and protecting the intestinal mucus barrier.^135^ Butyrate stimulates β-oxidation in mitochondria by activating the nuclear receptor peroxisome proliferator-activated receptor (PPAR) in colonocytes, which maintains anaerobic conditions in the intestinal lumen.^136^ SCFAs also decrease the pH of the intestinal lumen and prevent the growth of pathobionts.

Collectively, these acids serve as an additional energy source and concurrently regulate various systemic processes by activating signaling cascades within the host. However, it is important to note that observational studies conducted on human obesity have often have frequently yielded disparate findings regarding the quantification of SCFAs in feces or blood. For instance, one study indicated that obese children and adolescents harbor a higher abundance of carbohydrate-fermenting bacteria in their microbiota compared to normoweight counterparts, thereby augmenting SCFAs biosynthesis rates and providing the host with an additional energy source, ultimately stored as lipids or glucose.^74^ This apparent paradoxical observation implies that in addition to the SCFAs levels made by some members of the gut microbiota, there might be a direct recognition of specific bacterial components by some GPCRs in the host, which in turn modulate other metabolic processes that may contribute to obesity. Furthermore, it is important to note that the physiological mechanisms of SCFAs have primarily been studied in animal models and require further validation through clinical trials.

DFs may also further influence clinical outcomes in obesity by modulating the gut microbiota, thereby affecting the metabolism of the bile acid pool in the gut. Bile acids (BAs) are cholesterol metabolites produced in hepatocytes and specific gut microbes, such as *Lactobacillus*, *Bifidobacterium*, and *Bacteroides*, that can metabolize into secondary BAs through processes of depolymerization, dehydroxylation, and repolymerization.^137^ Disturbances in the gut microbial population prevent the conversion of primary bile acids, leading to their accumulation and a decrease in secondary bile acids. Primary bile acids act more specifically on G protein-coupled receptors (TGR5), whereas secondary bile acids primarily act on the Farnesoid X receptor (FXR).^138^ The activation of these receptors has been shown in animal models to cause changes in lipid and carbohydrate metabolism, energy expenditure, and inflammation.^137^

### 3.2. Role of dietary fibers in the management of the gut microbiome in children with obesity

As mentioned above, there is evidence that children with obesity have unique gut microbiota signatures that differ from that of healthy children.^21,139^ Therefore, whether nutritional interventions can redress microbial imbalances in pediatric obesity has garnered increasing attention in the field of nutrition. It is commonly recognized that a healthy diet is a critical environmental factor mediating the composition and metabolic function of the gut microbiota. DFs modulate the gut microbiome primarily including three strategies: 1) with more complex dietary patterns that provide rich sources of intrinsic DFs; 2) with a symbiotic mixture of live microorganisms and selective prebiotics fiber(s); 3) with the isolated fiber(s). The impact of diet on the composition of the gut microbiota is summarized in Table 1.

**Table 1.** Studies in which the effects of different DFs on gut microbiota in children with obesity was assessed.

	Trial design	Study Population	Dose	Age	n	Duration	Methodology	Microbiota	Microbial Diversity	Metabolic Effects	References
**Intrinsic DFs and high-fiber diet pattern**											
Low fruit and rich in meat	Cross-sectional study	Patients with Prader – Willi syndrome (overweight or obesity)	23.1 ± 13.4 g/day	5-8	31	4-day food diary	16S rRNA	*Klebsiella↑* *Eubacterium↑* *Lactobacillus↑* *Lachnoclostridium↓ Murimonas*↓ *Alistipes*↓ *Prevotella↓*	Shannon index*↑*	*Klebsiella* are significantly positively correlated with total and LDL-cholesterol, *Alistipes* are significantly negatively correlated with body fat mass, total and LDL-cholesterol levels, fasting insulin levels and HOMA-IR.	Garcia-Ribera et al^140^
51.6% total carbohydrates, 21.9% total sugar, 5.1% fiber, 31.8% total fat, 16.5% total protein	Cross-sectional study	Overweight or Obesity	16.1 ± 6.7 g/day	12-19	52	24-hour diet recalls	16S rRNA	*Bacteroides↑* *Prevotella↑* *Ruminococcus↑* *Blautia↑*	none stated	Fructose intake is negatively associated with the genera *E. eligens* and *Streptococcus thermophilus*	Jones et al^141^
High carbohydrate/high fat diet or high protein/high fat diet	Prospective study	Overweight or Obesity	None stated	2-9	70	7-day diet recalls	16s rRNA	*Bacteroides*↑ *Roseburia*↑ *Lachnospira*↑ *Slackia*↓ *Prevotella*↓ *Oscillospira*↓	none stated	Inflammatory (IL-15,TNF-α, IP-10,IL6,IL-8)↑	Rampelli et al^142^
Higher fiber and nonstarch polysaccharides diet	Cross-sectional study	Overweight or Obesity	18 g/day	5	319	one months food diary	16S rRNA	*Faecalibacterium↑ Eubacterium↑* *Roseburia↑*	Shannon index*↑*	None stated	Leong et al^143^
High traditional vegetable-based, low fat, sugar diets	Cross-sectional study	28 rural children (Buriram)	None stated	9-11	45	7-day dietary records	16S rRNA	Lachnospiraceae*↑* Peptostreptococcaceae↑	ACE index*↑* Chao1 index*↑*	Parabacteroides↑ Butyrate↑	Kisuse et al^144^
Whole grains, traditional Chinese medicinal foods, and prebiotics diet	Dietary intervention trial	Prader – Willi syndrome and obesity	50 g/day	3-16	36	0,30,60,90 days	Metagenomic sequencing	*Faecalibacterium↑ Bifidobacterium↑* *Clostridium↑* *Collinsella↑* *Bifidobacterium catenulatum↑* *B.longum↑* *B.breve↑* *Bacteroides↓* *Ruminococcus↓* *Blautia* ↓ *Escherichi*a↓	None stated	None stated	Li et al^145^
					38			*Bifidobacterium spp.↑* *B. pseudocatenulatum↑*	Chao1 index*↓* Observed OTU*↓* Phylogenetic diversity*↓* Shannon index*↓* gene richness*↓*	Body weight↓ BMI↓ALT↓ AST ↓	Zhang et al^146^
Proteins and complex carbohydrate patterns	Cross-sectional study	Overweight or Obesity	None stated	6-12	46	\	Metagenomic sequencing	*Holdemania spp.↑* *Coprococcus catus↓*	none stated	z-BMI↓ waist circumference↓ hip circumference↓	Orbe-Orihuela et al^147^
Whole grains, traditional Chinese medicinal foods, and prebiotics diet	Dietary intervention trial	Overweight or Obesity	50 g/day	3-18	87	30, 60, 90 days	16S rRNA	*Bifidobacterium↑* *Lactobacillus↑*	none stated	None stated	Hou et al^68^
**Synbiotic**											
Synbiotic (FOS）	Randomized triple-masked controlled trial	Obesity	one capsule	6-18	70	8 weeks	surface-cultured of plates	*Lactobacillus*↑ Bifidobacteria↑	none stated	Weight↓ Body mass index↓ Z-score↓ IL-6↓ TNFα↓ Hs-CRP↔ Adiponectin↑	Kelishadi et al^140^
Synbiotic (FOS）	Single-center, prospective, randomized, double-blind, placebo-controlled clinical study	Obesity	1 sachet each day	8-17	54	12 weeks	16S rRNA	Compared to baseline: Bacteroidetes↑ *Prevotella*↑*Dialister*↑ F/B ratio↓ Compared to placebo groups: *Collinsella stercoris* species were dominant in the synbiotic group	Chao1 index↔ Observed ASV↔ Simpson index ↔ Shannon index↔	None stated	Kilic Yildirim et al^148^
**Isolated fiber**											
Inulin	Double-Blind, RCT	Obesity	8 g/day	7-12	42	16 weeks	16S rRNA *qPCR*	Actinobacteria↑ *Bifidobacterium*↑ *Collinsella*↑ *B. adolescentis*↑ *B.longum*↑ *Ruminococcus↓* *F.prausnitzii↓* *E. eligens↓* *B.vulgatus↓* *R.gauvreauii↓*	Shannon index↑ Simpson index ↔ observed OTUs↑ beta-diversity (*p* > 0.05)	Body Weight↓ Body Fat (%)↓ trunk fat ↓ IL-6↓ TAG↓ primary bile acids↔	Nicolucci et al^149^

#### 3.2.1. Effects of intrinsic dietary fibers and high-fibers diet pattern on the gut microbiota of children with obesity

As depicted in Figure 1, Actinobacteria, *Prevotella*, *Bifidobacterium*, *Dialister*, *Escherichia*, *Anaerostipes*, *Akkermansia*, *Bacteroides* and α-diversity metrics other than the Simpson index showed a tendency to be less prevalent in children with obesity, and although there was no significance, inclusion in the Meta-analysis reported low heterogeneity. In high DFs dietary patterns 11% (1/9) of studies reported an increase in the Shannon index, ACE index and Chao1 index and 11% of studies (1/9) reported a decrease in Chao1 index, Observed OTUs, Phylogenetic diversity and Shannon index. An increase in *Prevotella* is associated with long-term dietary patterns, complex carbohydrates, or plant-based foods.^150^ Many of the species that respond to changes in traditional diet intake (high plant-based and low-fat foods) seem to belong to Actinobacteria, which are nutritionally specialized.^151,152^ Actinobacteria (such as *Bifidobacterium*, *Bifidobacterium* spp.) few genera increase in response to high-fiber diets.^143,145–147^ In response to the Western diet, the abundance of *Bacteroides* and *Klebsiella* increased, which was accompanied by a decrease in *Prevotella*,^141 ,142 ,153^

Recently, there has been an increasing recognition that consuming DFs naturally present in food (in contrast to consuming isolated DFs as supplements) may be most effective at regulating gut bacteria and enhancing overall health.^154^ Regarding dietary interventions for childhood obesity, it is important to consider not only whether the gut microbiota is being modulated, but also the improvement of metabolism in children. SCFAs, as key metabolites of fermentation of DFs by the gut microbiota, have a broad impact on various aspects of host physiology,^155^ Table 1 shows that only one cross-sectional study investigating high-fiber diets provided evidence of increased SCFAs.^144^ A traditional grain diet improved weight, BMI, and liver function metabolism in children with obesity, according to another study.^146^ It is important to note that while the effects of high-fiber diets on the body’s metabolism should be examined in relation to the microbiota, these diets may also affect the metabolism directly, independent of the microbiota. Foods such as those replaced by DFs (if they replace monosaccharides) produce additional benefits.

It is tempting to target ecological markers similar to those found in children with healthy diets (higher richness and diversity, stimulation of beneficial microbes), but it is unclear which microbiota response in children should be supported. In conclusion, most studies have shown a strong association between dietary patterns, gut microbiome, and childhood obesity.

#### 3.2.2. Effects of synbiotics mixture (live microorganisms and selective prebiotics fibers) on the gut microbiota of children with obesity

Synbiotic mixtures are a combination of prebiotics and probiotics that may be used to moderate dysfunctional gut microbiomes.^156^ The effect of synbiotics on α-diversity was not observed in the included studies (only 1 of the 2 studies reported relevant results). Kelishadi^140^ and colleagues used synbiotics capsules (fructooligosaccharides [FOS] plus *Lactobacillus* and Bifidobacteria) to intervene in children with obesity for eight weeks and found significant increases in *Lactobacillus*, as well as significant reductions BMI, IL-6, and tumor necrosis factor-α (TNFα). Another study used a Synbiotics formulation (probiotics mixture including *Lactobacillus acidophilus*, *Lacticaseibacillus rhamnosus*, *Bifidobacterium bifidum*, *Bifidobacterium longum*, *Enterococcus faecium* (total 2.5 × 10^9^ CFU/sachet) and FOS (625 mg/sachet) in children with obesity over a 12-week period; compared to baseline, Bacteroidetes, *Prevotella*, and *Dialister* significantly increased, F/B ratio and BMI significantly decreased.^148^ Interestingly, Bacteroidetes was significantly decreased and *Dialister*, *Lactobacillus* showed a trend of reduction in children with obesity. These data suggest that synbiotics can drive changes in the gut microbiota of obese children, with the potential to increase the diversity of the gut microbiota. However, synbiotics formulations often require higher than expected dosages of prebiotics for practical use to ensure that substrates are available for both the resident microbiota and the co-administered microbes in a highly competitive microenvironment within the gut.^157–159^ Therefore, Precise understanding of the synergistic impact of probiotics and prebiotics in synbiotics, as well as the mechanism by which they regulate the gut microbiota in children with obesity, remains challenging within the realm of precision nutrition.

#### 3.2.3. Effects of isolated dietary fibers on the gut microbiota of children with obesity

Isolated DFs can have a significant impact on the composition, diversity and abundance of the microbiome, providing a wealth of substrates for fermentation reactions carried out by specific microbial species.^160^ Microorganisms possess the necessary enzymes to hydrolyze a wide variety of complex carbohydrates, including glycoside hydrolases, glycosyltransferases, polysaccharide lyases, and carbohydrate esterases.^161^ As a result, having a variety of DFs (e.g., cellulose, inulin, fructans, resistant starches) in a diet that contains a range of monosaccharide units and α and β linkages is more supportive of a diverse gut microbial community than a diet with a less diverse substrate load (e.g., refined diets).^162^ Despite the growing body of literature on the ability of isolated DFs to positively improve childhood obesity,^163,164^ there is limited information on their impact on microbial diversity and abundance. Several studies have demonstrated that the intake of isolated DFs shapes the gut microbiota of children with obesity.^149,165^ In children with overweight or obesity, 16 weeks of oligofructose-enriched inulin (8 g/day) increased α-diversity (Shannon index, Observed OTUs), associated with an increase in Actinobacteria (such as *Bifidobacterium*) and a decrease in Firmicutes (such as *Ruminococcus*).^149^ Meanwhile, at the level of OTUs, *F. prausnitzii* decreased significantly. These changes in microbiota outcomes suggest that DFs are better at improving gut microbiota in obese children (Figure 2). Moreover, this prebiotics intervention improved children’s body weight, body fat, IL-6 levels, and triglyceride content. These beneficial effects of isolated DFs on childhood obesity aspects are thought to be at least partially mediated by the microbiota induced changes but more direct evidence still has to be provided.

In summary, intrinsic dietary, synbiotics, and isolated fibers exert specific modulatory effects on the gut microbiota composition and α-diversity in children with obesity. Current evidence does not support that DFs can modulate the bacteria (e.g., *Akkermansia, A. muciniphila*) found to be significantly decreased in children with obesity in the systematic review. *A. muciniphila*, a mucin-degrading bacterium, has a negative association with obesity, diabetes, and colitis.^166^ Supplementation with oligofructose has been shown to increase the number of goblet cells in animals, which subsequently exerts a prebiotic function on *A. muciniphila*.^167^ High-fermentable (pectin) fiber also modulates the gut immune environment in mice, which in turn promotes *Akkermansia* enrichment,^168^ and the findings suggest that the fiber effect on *Akkermansia* may be independent of fermentation but rather by improving the gut microecological environment. Furthermore, low-fermentable fibers augment crypt length, goblet cell maturation, and mucin secretion via mechanical stimulation caused by increased stool bulk.^169^ Cellulose (an insoluble fiber with low-fermentable) supplementation significantly increased *Akkermansia* abundance in mice with colitis, potentially by promoting mucin secretion from goblet cells.^170^ Low-fermented fibers effectively accelerate gut transit time, which may have implications for regulating the structure of the gut microbiota.^171^ However, there is a lack of clinical evidence investigating the impact of DF supplementation on *Akkermansia* in children with obesity, particularly the effect of isolated low-fermentable fiber on gut microbiota in this population.

#### 3.2.4. Dietary fibers and gut microbiota in children with obesity: remaining unsolved questions

The identification and implementation of suitable nutritional intervention techniques for modulating the gut microbiota in children with obesity is a prominent subject of interest for forthcoming scientific investigations. For many years, the study of DF-microbiota interactions using a reductionist approach has elucidated DFs dependent and independent effects on specific microbiota.^172^ Currently, dietary patterns with high DFs are an important strategy in nutritional intervention programs. As shown in Table 1, the more complex dietary interventions assessing the effect of DFs on the gut microbiota in children with obesity provide relatively more evidence, but substantial evidence is still lacking in general (such as randomized clinical trials). Further investigation is required to ascertain the fundamental mechanisms responsible for the distinct impacts of isolated fibers and intrinsic fibers on the gut microbiota of children with obesity.^173^ In addition, DFs have a significant impact on the composition, diversity, abundance, and function of the gut microbiota in children with obesity, but the strength of the evidence remains insufficient. As shown in Figure 1 and Figure S1, there is a clear downward trend in Bacteroidetes, *Prevotella* and *Bacteroidetes* among children with obesity. Not surprisingly, following a high DF intake, all of these bacteria in children with obesity moved in a favorable direction (Table 1). There is still a large degree of uncertainty about the extent to which the effects attributed to DFs on obesity are mediated by the gut microbiota in children and the key species involved in the effects. However, current evidence still does not support the extent to which the effects of DFs on obesity are mediated by the gut microbiota in children and the key species involved in the effects.

However, there is no unanimous agreement on the recommended amounts of fiber for children with obesity, and there is a lack of evidence regarding practical advice on the appropriate type and quantity of DFs to be consumed. It is imperative to acknowledge that existing guidelines for DF consumption in children pertain to the overall intake of fibers and fail to sufficiently account for the origin and type of fibers.^174^ This is important because various types and sources of fibers exhibit distinct physiological effects. For instance, the source (natural or synthetic, etc.), chemical composition (chain length, lignification, etc.), and physicochemical properties (solubility, viscosity, fermentability, etc.) of DFs affect not only the gut microbiota but also health outcomes.^175,176^ Hence, in the formulation of a nutritional intervention program targeting children with obesity, it is imperative to take into account not only the overall daily consumption of DFs but also to acknowledge the origin and type of fiber duly. Furthermore, additional research is required to investigate the physiological characteristics of the association between DFs and the health of children with obesity, as well as the optimal ratios of the different fiber sources.

Furthermore, the mechanism of action of DFs in regulating the gut microbiota to ameliorate childhood obesity has not been fully explored. We should be aware that the gut microbiota is an extraordinarily mutually dependent community in which waste products from one organism become nutrients for other organisms via substrate and metabolite cross-feeding. The cross-feeding behaviors of primary degraders and cross-feeders result in a wide range of substrates and metabolites to support microbiota diversity, allowing the gut to maintain a stable community.^177^ For example, in vitro analysis revealed that the relationship between bifidobacteria and *F. prausnitzii*, in the presence of inulin-type fructans, could be commensal or competitive, and this relationship was dependent on the bifidobacterial strain and its capacity for prebiotics degradation.^178^ More importantly, the gut microbiome profile of children with obesity is complex, with changes in the fungi, viruses, and archaea organisms in addition to bacterial homeostasis. To date, no studies have systematically reported the effects of DFs on the fungi, viruses and archaea organisms in the gut of children with obesity. Therefore, we should further our understanding of the complex relationships between fibers, gut microbiota, and childhood obesity, which can lead to the development of low-cost, safe, and efficacious “microbially-directed” dietary interventions.

## 4. Future direction

**Figure 3.**
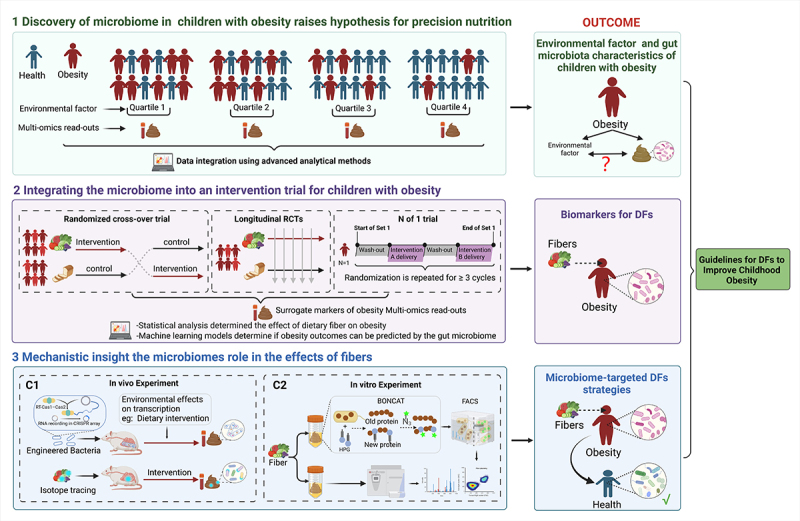
Experimental framework of the fiber-microbiota-obesity study in children. (a) microbiome epidemiology can elucidate associations between the gut microbiome, environmental factors (e.g., diet, age, sex, etc.), and physiologic effects caused by environmental factors in children with obesity. This information does not establish causality, but can help to uncover gut microbiome characteristics in children with obesity and contribute to the evidence base for precision nutrition. (b) RCT can determine the causal relationship between DFs and childhood obesity and provide strong evidence of this. N of 1 employs the “individual” as the subject of the study, allowing for the development of personalized and precise nutritional requirements. Combined with microbiome-based multi-omics analysis, microbial biomarkers for precision nutrition strategies can be identified. However, highly personalized microbiomes and causal inference of their role in DF’s effects still pose challenges. (c) causal relationships and potential mechanisms can be further established in in vivo (animal models) and in vitro studies. Causal and mechanistic information provides additional evidence for the development of dietary guidelines and targeted nutritional strategies for DFs. BONCAT, bio-orthogonal non-canonical amino acid tagging; CRISPR, clustered, regularly interspaced, short palindromic repeats; DFs, dietary fibers; FACS, fluorescence-activated cell sorting; RCT, randomized controlled trial.

Information on fiber-microbiome-obesity interactions in children needs to be further validated and refined. To be actionable, integration of the gut microbiome into the prevention of childhood obesity requires evidence of the causal contributions of the microbiome to obesity and the mechanistic physiological effects of fibers. Establishing the causal role of the gut microbiome in susceptibility to chronic diseases such as childhood obesity remains a challenge, which is complicated in nutrition research because the interactions between fibers, the gut microbiome, and human health are complex and multidirectional. These complexities must be considered in the design of future nutrition research to elucidate what factors, including the gut microbiome, mediate the effects of fibers in childhood obesity. We suggest extending future studies through an experimental framework using three pillars that integrate the gut microbiome into stages of fiber nutrition research on children with obesity (Figure 3).

### 4.1. Discovery of microbiome in children with obesity generates hypothesis for precision nutrition

Characterization of the gut microbiome may help in the prevention of childhood obesity and related metabolic diseases in the future. Furthermore, the application of multi-omics techniques (e.g., metagenomics, proteomics, metabolomics) and advanced analytical methods, such as machine learning-mediated analyses, has laid the groundwork for elucidating the underlying mechanisms and cause-and-effect relationships of biological pathways.^179,180^ Recent studies have expanded on this framework by combining the microbiome and the epidemiology of childhood obesity to elucidate the microbiological profile of children with obesity.^181^ Although such observational studies cannot establish causality, high-quality, large-scale cohort studies can help provide an evidence basis for the causes of childhood obesity. Integrating childhood obesity with microbiome epidemiology will facilitate the identification of links between the microbiome and childhood obesity and the characterization of the gut microbiota in children with obesity. The underlying mechanisms and biological plausibility of these interactions, as well as their value as diagnostic markers, can then be confirmed in experimental systems to confirm the system.

### 4.2. Integrating the microbiome into nutritional intervention trials in childhood obesity

More clinical research using fiber interventions in childhood obesity, aimed at addressing the gut microbial perspective is needed, as the number of studies identified thus far is small, as there is a paucity of data available. Notably, although numerous epidemiological studies have demonstrated an association between DF intake and childhood obesity, there have been some inconsistent reports. In contrast, a clinical trial by Liber and Szajewska^182^ showed that consumption of 8 g/d of oligofructose in otherwise healthy overweight or children with obesity aged 7–11 years and 15 g/d in children aged 12–18 years did not result in improvements in BMI or Z-score after 12 weeks compared with placebo control. There were also no improvements in the secondary outcomes of change in body weight and difference in absolute body fat or other metabolic outcomes. A systematic review and meta-analysis of the relationship between DFs and diabetes management found similar results.^183^ These findings point to variation in the response of DFs to obesity-related metabolic dysregulation in children, which is thought to be due to the high degree of individualization of gut microbes in children. The research of Deehan et al. supports this theory.^184^ A DF intervention is likely to have no effect on an individual whose microbiome does not include keystone species or other genera that encode the enzymatic machinery to degrade those specific fiber types. This should thus be considered when designing future studies.

Randomized controlled trials are the gold standard for establishing causal effects. Optimally, feeding studies with single ingredient modifications using crossover designs with sufficient washout periods are ideal to assess not only the impact of DFs on gastrointestinal bacteria taxa, but also microbial metabolites and other physiological measures of health such as body composition, blood cholesterol, glycemia, and inflammation. It is critical to include controls, which can eliminate inter-individual differences in individual-specific factors (e.g., microbiome, genetics, metabolite profiles, and baseline clinical measures).^185^ Furthermore, for the highly individualized gut microbiome of children with obesity, a precision nutrition research design (N of 1 trial) can be used to screen for nutritional interventions that are appropriate for them, using themselves as a control, to achieve true, albeit difficult, precision nutrition.^186^

Studies should account for variation in other confounding variables such as demographics (age^187^ and gender^188^) and lifestyle factors (such as habitual diet).^189^ Obviously, in view of the complexity of the confounding variables, we need advanced statistical and modeling methods to carry out data analysis. For example, regression and correlation analyses can be applied to determine associations between fibers-induced changes in microbiome composition/functionality and clinical and mechanistic endpoints.^190^ Machine-learning models can determine if fibers-induced physiological changes can be predicted by effects on the microbiome or biological processes in the host impacted by the microbiome.^191^ Integration of the microbiome into human intervention trials could provide putative mechanistic explanations for the role of the microbiome in the effects of fibers on childhood obesity, as well as diagnostic microbiome-based biomarkers for precision nutrition strategies.

### 4.3. Mechanistic insight the microbiomes role in the effects of fibers

Human studies can be paired with animal models to determine whether the microbiome plays a causal role in the physiological effect, identify the causal components within the microbiome, and determine underlying mechanisms^192^ (Figure 3 C1). The complex and dynamic transcriptional changes of the gut microbiota throughout time, transit, and perturbation in the gut of gnotobiotic animals are recorded by integrating RNA-derived spacers from the transcriptome of gut bacteria into plasmid DNA-encoded Clustered, regularly interspaced, short palindromic repeats (CRISPR) arrays using FsRT-Cas1-Cas2^193^ (Figure 3C1). This scalable, noninvasive system for assessing intestinal function in vivo archives characteristics of microbial signatures of physiological or pathological states.^177^ Transcriptome-scale recordings elucidate microbial responses to alterations in the intraluminal environment across nutrition and microbe-microbe interactions.^177^ Furthermore, a method for tracing isotopes into bacterial-specific protein sequences has been developed that enables the mapping of nutrient routes in vivo and reveals how diet affects the makeup of the microbiome (Figure 3 C1). However, mechanistic studies in animals can be confounded as it is not possible to focus solely on the gut microbiota due to the presence of the host.^194^ Noteworthy, in vitro gut fermentation models can be used to supplement human and animal studies and overcome some of the limitations of in vivo models^195^ (Figure 3 C2). Researchers can use a variety of in vitro models in which single or multiple vessels are inoculated with fresh human feces or a defined microbial community. These vessels are operated under anaerobic conditions, and microbial communities are grown with temperature, pH, growth medium, and transit time set to mimic a specific intestinal segment. This tool can be used in combination with new technologies (e.g., Bio-orthogonal non-canonical amino acid tagging-Fluorescence-activated cell sorting [BONCAT-FACS],^196^ Figure 3 C2) to test the direct effects of prebiotics and DFs on the gut microbiota of children with obesity in the absence of confounding factors. While both animal and in vitro models can mimic some aspects of the human gut microbiome, neither model can fully replicate all of the functions of the human gut. Despite their limitations, their microbiomes, if well controlled and combined with a multi-omics approach, have the capacity to complement human studies as they establish the mechanistic basis for the impact of fibers on children with obesity.

## 5. Conclusion

The current evidences suggest that a higher intake of DFs may have a beneficial impact on metabolic health through modifications to the gut microbiota.^175^ Personalized fiber intake recommendations and improved obesity outcomes ought to be feasible with the integration of individual-level genetic and metabolic trait data, along with the development of microbial sequencing technologies and analytical methodologies. Microbiome research possesses the opportunity to furnish exhaustive insights into facets of nutrition strategy. However, the gut microbial signatures observed in children with obesity relative to normoweight children remain unclear and require further research. Overall, in order to improve the convincing evidence supporting dietary guidelines for children, it is imperative that facets of childhood obesity prevention research incorporate gut microbial features.

## Supplementary Material

Supplemental Material

## Abbreviations

BAs: Bile acids
BONCAT-FACS: Bio-orthogonal non-canonical amino acid tagging-Fluorescence-activated cell sorting
CRISPR: Clustered, regularly interspaced, short palindromic repeats
DFs: Dietary fibers
FOS: fructooligosaccharides
FXR: Farnesoid X receptor
GPCR: G protein-coupled receptors
GLP-1: glucagon-like peptide 1
HDL: high-density lipoprotein
LPS: Lipopolysaccharides
PPAR: peroxisome proliferator-activated receptor
PYY: peptide secretion YY
RT-PCR: Real-time PCR
SCFAs: short-chain fatty acids
TNFα: tumor necrosis factor-α
UCP2: uncoupling protein 2
VLPs: virus-like particles
BMI: body mass index
