# Supplementary material for: Dietary fiber for the prevention of childhood obesity: a focus on the involvement of the gut microbiota
Source: Gut Microbes. 2024 Aug 20;16(1):2387796. doi: 10.1080/19490976.2024.2387796 (PMC11340751; doi:10.1080/19490976.2024.2387796)
Supplement: Supplemental Material [file KGMI_A_2387796_SM5515.zip › Supplementary Materials.doc]

**Supplementary Materials**

**METHOD**

The protocol was registered through PROSPERO (Registration no. CRD42023408309) and data extraction followed the 2009 Preferred Reporting Items for Systematic Reviews and Meta-Analyses guidelines.

Eligibility Criteria

The PICOS (population, intervention, comparison, outcome and study design) framework was used to guide this systematic review. The eligible study designs include randomized or non-randomized trials, crossover, cross-sectional, parallel design, and prospective observational studies. The population of interest was children (3-18 years old) subjects with obesity and/or metabolic syndrome. Collection and analyses of the gut microbiome through fecal samples and at least one adiposity measure were permitted. Our primary outcomes of interest changeα-and β-diversity and gut microbiome composition. Studies with unavailable gut microbiome data were excluded. To ensure all relevant studies were identified and included, we also manually searched through reviews and publication references. This study was limited to original human studies without any language restrictions.

Search strategy

The databases PubMed, EMBASE, Scopus, Web of Science, Google Scholar, CNKI, Cochrane Library and Wiley online library were used to conduct a systematic literature search, as outlined in Box 1 Figure. The selected search terms were as follows: (obesity OR “metabolic syndrome” OR “metabolic disease” OR “metabolic disorder” OR obese) AND (“gut microbiota” OR “gut microbiome”) AND (child* OR adolescent* OR young* OR youth* OR kid OR teen*) NOT (mice OR cats OR rats OR rodent* OR animal* OR “guinea pig”). Titles and abstracts of relevant articles were first assessed by two independent reviewers (YZM and YMY). Studies meeting initial screening criteria by at least one reviewer were selected for full-text review. Two independent reviewers examined all full-text articles for eligibility (YZM and YMY). Disagreements were resolved through discussion and by the decision of a third reviewer when needed.

Data Extraction

Data was extracted independently by two reviewers (YZM and YMY) and discrepancies were resolved by consensus or through assessment by a third independent reviewer (CCX). Relevant data from all publications were extracted independently into an Excel document and cross-examined for accuracy. Study characteristics were evaluated for type of study design, year of study, and country of origin. Population variables included a number of patients, age, sex, gut microbial changes, donor stool processing, and delivery methods (see online supplement table 1).

Assessment of Risk of Bias

Methodological quality was assessed for each publication according to the Joanna Briggs Institute (JBI) Critical Appraisal of Evidence Effectiveness tool. Two reviewers (YZM and YMY) completed this assessment independently and all discrepancies were resolved through discussions (supplementary Table S1, S2 and S3). Publication bias was assessed via funnel plots when more than ten studies were included in the forest plot. Publication bias was not assessed when there were fewer than ten studies.

Data Synthesis and Analysis

The quantitative and qualitative data of gut microbiota diversity and relative abundance between obese and non-obese children reported in each study were recorded and were synthesized by qualitative summary and meta-analysis, respectively. For qualitative analysis, the results of each

prespecified outcome were summarized and presented as stacked percentage bar charts. For meta-analysis, standardized mean difference (SMD) and 95% confidence intervals (CIs) were calculated to evaluate the differences in diversity indices and relative/absolute abundances of gut microbiota between obese and non-obese children. Significance was set at *P*<0.05. A random-effects model meta-analysis was performed to calculate the pooled mean based on heterogeneity. *I^2^* statistic was used to assess the heterogeneity between the studies. In the case of *I^2^*≥50%, heterogeneity was explored further with sensitivity analyses. Sensitivity analysis and subgroup analysis were performed according to different inclusion criteria for obese children. All the statistical processes and results visualization were conducted by Review Manager (version 5.4), and GraphPad Prism (version 9.0).

**Supplemental Table 1: Study Characteristics**

| **Reference** | **Country** | **Study Population** | | | **Study Design** | **Microbiome assays** | **Microbial Abundance (obesity relative to control)** | | | **Microbial Diversity** | | **Structure and Activity of the Microbiota** |
| --- | --- | --- | --- | --- | --- | --- | --- | --- | --- | --- | --- | --- |
|  |  | **Sample Size** | **Age (years)** | **Composition** |  |  | **Phylum level** | **Genus level** | **Species level** | **α** | **β** |  |
| Borgo F et al.2017 | Italy | 61 | 8-12 | 28 obese  33 normal | Case-control study | 16S rRNA Real-time PCR | NA | *Bacteroides/Prevotella*↓ | *Bifidobacterium spp.**↔Lactobacillus spp.↔*  *Akkermansia muciniphyla ↓ Faecalibacterium prausnitzii ↓ Saccharomyces cerevisiae ↓ Candida albicans**↓* | NA | NA | NA |
| López-Contreras BE et al.2018 | Mexio | 138 | 6-12 | 71 obese  67 normal | Case-control study | 16S rRNA | Firmicutes/Bacteroidetes*↔* |  | *Bacteroides eggerthii*↑  *Bacteroides plebeius**↓*  *Faecalibacterium prausnitzii↔Bifidobacterium adolescentis↔Bacteroides caccae↔Bacteroides fragilis↔Bacteroides ovatus↔Akkermansia muciniphila↔Bacteroides uniformis↔* | Shannon index*↔*, Observed species*↔*, Richness index*↔* | NA | Christensenellaceae had a negative relationship with total cholesterol,Apo B, phenylalanine,arginine,methionine,ornithine and gamma glutamyl transpeptidase level.*Odoribacter* was adversely linked with insulin and homair levels. In addition, Citrulline levels and *B. eggerthii* were negatively connected, whereas *B. plebeius* and phenylalanine levels were negatively correlated. |
| Vazquez-Moreno M et al.2021 | Mexio | 330 | 6-12 | 163 obese  167 normal | Cross-sectional study | 16S rRNA | Firmicutes↑, Proteobacteria*↔*  Actinobacteria*↔*  Cyanobacteria*↔*  Tenericutes *↔*Verrucomicrobia*↓*  Bacteroidetes*↓* | *Fusicatenibacter*↑,*Romboutsia*↑  *Anaerostipes↔, Blautia↔*  *Intestinibacter↔,Alistipes↔*  *Bacteroides↔* | NA | Species richness*↔*, Inverse simpson*↔*, | *P*<0.05 | Firmicutes and Bacteroidetes phyla were positively and negatively associated with obesity. |
| Gyarmati P et al.2021 | USA | 83 | 5-12 | 23 lean 11overweight 15 class 1 obesity  17 class 2 obesity  17 class 3 obesity | Cross-sectional study | 16S rRNA | Proteobacteria↑  Firmicutes/Bacteroidetes*↔*Verrucomicrobia*↓* | NA | NA | Shannon index*↔* | NA | Correlation between Proteobacteria and Verrucomicrobia and BMI z-scores |
| Benítez-Páez A et al.2020 | Spain | 51 | 5-17 | 16 lean  20 obese 15 obese+IR | Cross-sectional study | 16S rRNA |  |  | *Clostridium disporicum↔Eubacterium hallii↔*  *Blautia luti↓*  *Blautia wexlerae↓*  *Terrisporobacter sp.↓* | NA | *P*<0.05 | *Blautia luti* and *Blautia wexlerae* exhibited negative correlation with TNF-α values. *Clostridium*. disporicum negative correlation with MCP-1 |
| Shin S et al.2020 | Korean | 46 | 5-13 | 22 obese  24 normal | Cross-Sectional Study | 16S rRNA | Firmicutes/Bacteroidetes↑  Firmicutes*↔*  Actinobacteria*↔*  Proteobacteria*↔*  Bacteroidetes*↓* | *Actinomyces* ↑, *Romboutsia*↑  *Weissella*↑,*Akkermansia↔*  *Bacteroides↓, Anaerofilum↓*  *Alistipes↓ ,Oscillibacter↓*  *Hydrogenoanaerobacteriumr↓ Citrobacter↓* | *Bacteroides ovatus↓* | Simpson index*↔*Shannon index*↔*phylogenetic diversity*↔*  ACE index*↔*  observed species*↔*  Chao1 index*↔*  *Jackknife estimate↔* | *P*<0.05 | Bacteroidetes was negatively correlated with BMI z -score, hs-CRP level, and fat intake.on the other hand,the Firmicutes positive correlations with the inflammatory markers hs-CRP and neutrophil count.In addition, Actinobacteria positive correlations with HOMA-IR , hs-CRP and neutrophil count. |
| Barczyńska R et al.2018 | Polish | 40 | 6-15 | 10overweights  10 obese  20 lean | Cross-Sectional Study | Fluorescence in situ hybridization  16S RNA | Firmicutes↑, Bacteroidetes*↓* | *Clostridium*↑ *Lactobacillus↔* *Bacteroides↓Prevotella↓*  *Bifidobacterium↓* | NA | NA | NA | NA |
| Méndez-Salazar EO et al.2018 | Mexio | 36 | 6-11 | 12undernourished  12 obese  12 normal | Cross-Sectional Study | 16S rRNA | Proteobacteria↑ Firmicutes/Bacteroidetes↑ Bacteroidetes*↓* | *Bilophila*↑ | NA | Chao1 index*↓*  Shannon index*↓* | NA | NA |
| Xu P et al.2012 | Kazakhsta | 175 | 7-13 | 91normal  62overweight  22obesity | Case-control study | Real-time PCR | Firmicutes/Bacteroidetes↑  Firmicutes *↔*  Bacteroidetes*↓* | NA | NA | NA | NA | Bacteroidetes and Bacteroidetes/Firmicutes ratio were negatively correlated with BMI |
| Bervoets L et al.2013 | Belgium | 53 | 6-16 | 26 obese  27 control | Cross-Sectional Study | culture  MALDlI-TOF MS  Real-time PCR | Firmicutes/Bacteroidetes↑ |  | *Lactobacillus spp.*↑  *Bacteroides.fragilis↔*  *Bacteroides.Ovatus↔*  *Bacteroides thetaiotaomicron* *↔* *Bacteroides caccae↔* *Bacteroides uniformis*↔Clostridium spp.↔  *Bacteroides vulgatus* ↓ | NA | NA | *Lactobacillus spp.* has a favorable relationship with plasma hs-CRP levels |
| Visuthranukul C et al.2022 | Thailand | 164 | 7-15 | 164Obese  45 Non-obese(PS:previously published data ) | Cross-sectional study | 16S rRNA | Proteobacteria ↑, Fusobacteria↑ Firmicutes*↔* Firmicutes/Bacteroidetes*↔* Bacteroidetes*↓*  Actinobacteria*↓* | *Blautia*↑, *Lactobacillus*↑  *Collinsella↔*  *Clostridioides↔*  *Bifidobacterium↓* | NA | Shannon index ↔Simpson index↔ Chao1 index↔ | *P*<0.05 | Actinobacteria showed the strongest correlations with metabolic profiles including HDL-C , FI, and HOMAIR. Firmicutes was not correlated with BMI z-score or any metabolic profiles.*Bifidobacterium* was positively correlated with HDL-C but negatively associated with body weight. *Lactobacillus* was significantly and positively associated with fat mass,fat mass index (FMI), body fat percentage, trunk FMI, and visceral fat area (VFA). |
| Maya-Lucas O et al.2019 | Mexico | 20 | 9-11 | 10 obese  10 normal | Cross-sectional study | Metagenomic shotgun sequencing | Firmicutes/Bacteroidetes↑ | NA | *Megamonas spp.*↑  *Bilophila wadsworthia*↑  *Anaerostipes hadrus*↓  *Clostridium citroniae*↓  *Coprococcus come*↓  *Streptococcus thermophilus*↓ *Veillonella parvula*↓  *Ruminococcus spp.*↓  *Dorea formicigenerans*↓  *Oscillibacter spp.*↓  *Bacteroides ovatus*↓  *Bacteroides xylanisolvens*↓  *Bacteroides salyersiae↓*  *Bacteroides faecis*↓ | Shannon index ↔Simpson index ↔Chao1 index ↔Observed richness index ↔ | NA | NA |
| Orbe-Orihuela YC et al.2018 | Mexico | 890 | 6-14 | 507 obese  383 normal | Cross-sectional study | Real-Time PCR | Firmicutes↑, Bacteroidetes↓ | NA | NA | NA | NA | Firmicutes were significantly positively correlated with TNF-α and BMI.Furthermore, a positive correlation of TNF-α levels with BMI in the low tertiles of Bacteroidetes being statistically significant. |
| Karvonen AM et al.2019 | USA | 502 | 3 | 146 overweight/obese  356 normal | Prospective cohort | 16 S rRNA | Firmicutes/Bacteroidetes*↔* Verrucomicrobia↓ | *Dorea*↑, *Ruminococcus*↓  *Akkermansia*↓  *Parabacteroides*↓ | NA | Shannon index*↔* richness index*↔* | NA | Firmicutes/Bacteroidetes ratio were not associated with overweight/obesity. High relative abundances of *Parabacteroidetes* (Bacteroidetes; Bacteroidales) and Peptostreptococcae U. (Firmicutes;Clostridiales) were inversely associated with overweight/obesity, and similar tendency were seen with *Ruminococcus* (Firmicutes; Clostridiales) and *Akkermansia* (Verrucomicrobia;Verrucomicrobiales).High relative abundances of Parabacteroidetes (Bacteroidetes; Bacteroidales) and Peptostreptococcae U. (Firmicutes;Clostridiales) were inversely associated with overweight/obesity, and similar tendency were seen with *Ruminococcus* (Firmicutes; Clostridiales) and *Akkermansia* (Verrucomicrobia;Verrucomicrobiales) |
| Balakrishnan B et al.2021 | USA | 60 | 6-10 | 43 non-obese 17 obese | Cross-sectional study | 16 S rRNA | Actinobacteria↑  Proteobacteria↑  Firmicutes↑ | *Klebsiella*↑  *Megasphaera*↑  *Aggregatibacter*↑  *Eikenella*↑ |  | observed species↑ | *P*<0.05 |  |
| Del Chierico F et al.2018 | Italy | 69 | 13-19 and  20-65 | 25 obesity adolescents  20 obesity adult  12 normal adolescents  12 normal adult | Case Control study | 16 S rRNA | Actinobacteria↑  Firmicutes↑  Firmicutes/Bacteroidetes↑  Bacteroidetes↓ | *Actinomyces*↑, *Adlercreutzia*↑  *Streptococcus*↑, *Oscillospira*↓ | *Collinsella aerofaciens*↑  *Faecalibacterium prausnitzii*↑  *Propionibacterium acnes*↑  *Veillonella parvula*↑  *Bacteroides caccae*↓ | ChaoI index↓  Shannon index↔ | *P*<0.05 | Erysipelotrichaceae negatively correlated with the BMI. |
| Del Chierico F et al.2021 | Italy | 106 | 9-18 | 68 obesity  38 normal | Cross-sectional study | 16 S rRNA |  | *Dorea*↑, *Streptococcus*↑  Blautia↑, *Bacteroides*↓  *Oscillospira*↓  *Parabacteroides*↓ | *Bacteroides ovatus*↓ | Chao1 index↔Observed species↔ | NA | We found significant correlations between BMI Z-score, Actinobacteria, Lachnospiraceae, *Granulicatella,* Bacteroidetes , Bacteroidaceae, and *Odoribacter*. Significant correlations were also found between WBCs and Gemellaceae; DBP and *Dialister* ; HDL-C and *Clostridium*, *Turicibacter* and Gemellaceae; HOMA-IR and Clostridiaceae, *Coprococcus* and *Haemophilus* *parainfluenzae*; IGT and *Clostridium*. |
| Nirmalkar K et al.2018 | Mexio | 172 | 6-18 | 49 normal weight children  62 obese children  27 normal weight adolescents 34 obese adolescents | Cross-sectional study | 16 S rRNA | Firmicutes *↔*  Actinobacteria *↔*  Bacteroidetes *↔* | *Lactobacillus*↑, *Clostridium*↑  *Succinivibrio*↑, *Portiera*↑  *Dickeya*↑, *Prevotella*↑  *Blautia*↑, *Lactococcus* ↑  *Adlercreutzia*↑, *Gemella*↑  *Coprococcus*↑, *Collinsella*↑  *Stenotrophomonas*↑  *Bradyrhizobium*↑  *Bifidobacterium*↑  *Propiocinimonas*↑  *Streptophyta*↑  *Fusobacterium*↓ | NA | observed species↔  Chao1 index↔  Shannon index↔  Simpson index↔ | *P*>0.05 | Veillonellaceae significantly positively correlated with VCAM-1.*Ruminococcus*,*Oscillospira* significantly positively correlated with ICAM-1.In contrast, obese adolescents had a positive association between total cholesterol and *Ruminococcus*, and between ICAM-1 and Bacteroides (*P* = 0.0001, q = 0.102). Finally, there was a negative association between LDL and *Parvimonas.* |
| Riva A et al.2017 | Italy | 78 | 9-16 | 36 normal  42 obese | Prospective cohort | 16 S rRNA | Firmicutes ↑ Firmicutes/Bacteroidetes↑  Actinobacteria*↔* Verrucomicrobia*↔* Proteobacteria*↔*  Bacteroidetes↓ | *Subdoligranulum* *↔* *Faecalibacterium* *↔*  *Dialister↔, Blautia* *↔*  *Bifidobacterium* *↔* *Pseudobutyrivibrio↔*  *Bacteroides*↓ | *Bacteroides vulgatus*↓ | Observed species↔ Chao1 index ↔  Shannon index↔inverse Simpson↔ |  | The abundance of Firmicutes,*Faecalibacterium prausnitzii* and Ruminococcaceae were positively connected with BMI z-score, while Bacteroidetes were adversely correlated with it (as well as Bacteroidaceae and *Bacteroides* and *Bacteroides stercoriszspp.*). Furthermore,Faecalibacterium was either favorably connected with acetate and butyrate levels, and some genera within the Bacteroidetes were negatively correlated with acetate levels.Propionate levels were positively correlated with *Prevotella copri*. |
| Yuan X et al.2021 | China | 65 | 5-15 | 40insulin sensitive  25insulin resistance | Cross-sectional study | 16 S rRNA | Bacteroidetes ↑, Firmicutes ↓ Firmicutes /Bacteroidetes↓ | *Adlercreutzia* ↓  *Anaerostipes*↓  *Dialister*↓  *Haemophilus* ↓ *Turicibacter* ↓ | NA | Shannon index↔  Observed species↔ phylogenetic diversity↔ Pielou's evenness↔ | *P*<0.05 | Adropin positively correlated with genus *Anaerostipes* and *Alistipes*, and negatively associated with genus *Blautia*,and ANGPTL4 negatively correlated with the F/B ratio and genus *Bacteroides*, *Butyricimonas* and *Alistipes*. |
| Ferrer M et al.2013 | Spain | 2 | 15 | 1Lean  1Obese | Cross-sectional study | 16 S rRNA | Firmicutes ↑, Actinobacteria↑  Bacteroidetes↓ | *Bacteroides*↑, *Eubacterium*↑ *Prevotella* ↓ *Bacteroides/Prevotella*↓ | NA | NA | NA | NA |
| Goffredo M et al.2016 | USA | 84 | 14 | 15lean 7overweight  27obese  35Severely obese | Cross-sectional study | qPCR | Actinobacteria ↑ Firmicutes/Bacteroidetes↑  Bacteroidetes↓ | *Actinomyces*↑, *Bifidobacterium*↑  *Streptococcus*↑, *Blautia*↑  *Prevotella*↑, *Odoribacter*↓  *Oscillospira*↓, *Bacteroides*↓  *Faecalibacterium*↓ | NA | NA | NA | F/B, Bacteroidetes, and Actinobacteria were associated with visceral fat, SC fat and hepatic fat content. In addition,. F/B and Bacteroidetes were associated with acetate and propionate levels. Among the examined genera associated with BMI, *Actinomyces, Odoribacter, Bacteroides*, and *Faecalibacterium* showed a statistically significant association with all SCFA plasma concentrations as well, whereas *Oscillospira*, *Streptococcus*, and *Blautia* were associated only with acetate levels. |
| Grigorova EV et al.2021 | Russia | 40 | 11-17 | 22control  18obesity | Case-control study | 16S rRNA | Bifidobacteria ↓ | *Klebsiella*↑ *Enterobacter*↑  *Citrobacter*↑ *lactoflora* ↓ *Lactobacillus* ↓ | *Staphylococcus aureus*↑  *Enterobacter cloacae*↑  *Clostridium spp.*↑  *Enterobacter aerogenes*↓ | NA | NA | NA |
| lgnacio A et al. 2016 | Brazil | 84 | 3-11 | 30obese 24overweight  30 lean | Cross-sectional study | Culture  Real-time PCR  16S rRNA | NA | NA | *Lactobacillus spp* ↑  *Bacteroides fragilis* ↑  *Clostridium sp* ↑  *Bifidobacterium infantis* ↑  *Escherichia coli* *↔* *Bifidobacterium spp.*↓ | NA | NA | *Bifidobacterium spp.* was found to be negatively related to BMI.Moreover,*B. fragilis* or *Lactobacillus spp.* showed a substantial positive connection with BMI. |
| Abdallah Ismail N et al. 2011 | Egyptian | 79 | NA | 51 were obese (23 children and 28 adults), and 28 were normal weight (17 children and 11 adults) | Case-control study | PCR | Firmicutes↑  Bacteroidetes↑ | NA | NA | NA | NA | High fat intake and higher hsCRP were significantly associated with Firmicutes.Moreover，Bacteroidetes and Firmicutes were found to have the highest carbohydrate intake. |
| Jaimes JD et al. 2021 | Czech Republic | 52 | 7-16 | 16normal  17overweight  19obese | Cross-sectional study | 16 S rRNA | Firmicutes/Bacteroidetes↓ | Escherichia↓ | NA | Chao1index↔  Simpson index↔Shannon index↔ ACE index↔ | NA | NA |
| Karlsson CL et al. 2012 | Sweden | 40 | 4-5 | 20overweight/obese 20control | Case-control study | 16S rRNA  qPCR | NA | Lactobacillus *↔* Bifidobacterium *↔* Desulfovibrio↓ | B. fragilis*↔* Akkermansia muciphilialike ↓ | Simpson index ↔  Shannon index ↔ | NA | *Bifidobacterium* and ALT were found to be negatively related. |
| Murugesan S et al. 2015 | Mexio | 190 | 9-11 | 81normal 29overweight  80obese | Cross-sectional study | 16 S rRNA | Firmicutes*↔*  Proteobacteria*↔*  Actinobacteria*↔*  Bacteroidetes *↔* | *Faecalibacterium* ↑ | *Roseburia sp.* ↑, *Blautia sp.* ↑  *Coprococcus sp.* ↑  *Faecalibacterium sp.* ↑ *Succinivibrio sp.* ↓ *Erwinia sp.* ↓ *Oscillospira sp.*↓ | NA | *P*>0.05 | NA |
| Chen X et al. 2021 | China | 100 | 6-11 | 30 normal  35 overweight  35 obese | Cross-Sectional Study | 16S rRNA | Proteobacteria ↑  Fusobacteria ↑Bacteroidetes↓  Actinobacteria ↓  Tenericutes↓ | *Prevotella* ↑, *Sutterella*↑  *Megamonas*↑, *Fusobacterium* ↑ *Haemophilus*↑, *Sneathia*↑  *Veillonella*↑, *Aggregatibacter* ↑  *Megasphaera* ↓,*Clostridium*↓  *Oxalobacter*↓,*Desulfovibrio* ↓  *Christensenella* ↓  *Collinsella* ↓,*Anaerotruncus* ↓  *Holdemania*↓ | NA | Chao1 index ↓  observed species ↓  phylogenetic diversity↓  Shannon index ↔ Simpson index↔ | NA | NA |
| Chen X et al. 2020 | China | 51 | 6-11 | 23 normal  28 obesity | Case control study | 16S rRNA | Firmicutes*↔*  Firmicutes/Bacteroidetes*↔*Bacteroidetes↓ | *Faecalibacterium*↑ *Phascolarctobacterium*↑ *Lachnospira*↑ *Megamonas*↑  *Haemophilus*↑*Oscillospira*↓  *Dialister*↓ | NA | Chao1 index ↓  Shannon index↓  Observed species↔  Phylogenetic diversity↔ | *P*<0.05 | NA |
| Pierri L et al. 2018 | Italy | 16 | 8-13 | 10 obesity  6 normal | Cross-Sectional Study | 16 S rRNA | Bacteroidetes↑  Proteobacteria*↔*  Actinobacteria*↔*  Firmicutes↓ | *Bacteroides*↑*Prevotella*↑ | NA | NA | NA | Intestinal permeability were correlated with *Prevotella*,endogenous ethanol and systemic endotoxin serum values were significantly correlated with Proteobacteria and Gammaproteobacteria. |
| Cho KY et al. 2018 | Korean | 36 | 7-18 | 17 fat loss 19 fat gain | longitudinal cohort study | 16S rRNA | Bacteroidetes↓ | *Blautia*↑, *Dorea*↑ *Fusicatenibacter*↑ *Bacteroides*↓,*Oscillibacter*↓  *Parabacteroides*↓ | *Eubacterium hallii*↑ | Shannon index↓ observed species↓ | *P*<0.05 | NA |
| Yuan X et al. 2021 | China | 86 | 5-15 | 42 metabolic healthy obese 23 metabolic unhealthy obese  21 healthy normal weight controls | cross-sectional study | 16S rRNA | Synergistetes↑ Tenericutes↓ | *Fusobacterium*↑ *Bacteroides*↑, *Dorea*↑ *Pyramidobacter*↑  *Anaerostipes*↓, *Alistipes*↓ *Desulfovibrio*↓, *Oscillospira*↓  *Fusobacterium*↓ *Gemmiger*↓, *Odoribacter*↓  *Parabacteroides*↓ | NA | Shannon index↓ Chao1 index↓ | *P*<0.05 | *Lactococcus* positively correlated with IL-6.TNF-α positively correlated with Bacteroidetes, negatively correlated with *Citrobacter.*LBP positively correlated with *Prevotella, Odoribacter,* and negatively correlated with *Bifidobacterium, Streptococcus, Roseburia, Clostridium* and *Veillonella.* Leptin positively correlated with *Eubacterium* and negatively correlated with *Faecalibacterium* and *Lachnospira.* |
| Wei Y et al. 2021 | China | 236 | 6-9 | 60obese/overweight  120normal  56underweight | cross-sectional study | 16 S rRNA | Firmicutes/Bacteroidetes*↔* | *Blautia*↑, *Romboutsia*↑ *Anaerostipes*↓,*Clostridium*↓ | Ruminococcus gnavus ↓ Flavonifractor plautii ↓ | ACE index ↔ Chao1 index↔Shannon index ↔Simpson index ↔ | *P*>0.05 | *Ruminococcus* gnavus and *Flavonifractor plautii* showed significant negative correlations with the total and regional body fat , whereas *Blautia* and *Romboutsia* exhibited positive correlations with body fat measures |
| Balamurugan R et al. 2010 | India | 28 | 11-14 | 15 obese  13 non-obese | case-control study | Real-time PCR | NA | *Bacteroides/Prevotella*↔ *Bifidobacterium*↔ | *Faecalibacterium prausnitzii*↑  *Eubacterium rectale*↔  *Lactobacillus acidophilus*↔ | NA | NA | NA |
| Mousavi S H et al. 2008 | Iran | 188 | 7-12 | 60 lean  63 normal  65 obese | Cross-sectional study | Real-Time PCR | Firmicutes↑ Bacteroidetes↓ Firmicutes/Bacteroidetes↑ | NA | NA | NA | NA | bact/firm ratio is significantly negatively correlated with BMI and waist |
| Gallardo-Becerra L et al. 2020 | Mexio | 27 | 7-10 | 7 normal weight  10 obesity  10 obesity and metabolic syndrome | Case-control study | 16 S rRNA | Firmicutes/Bacteroidetes ↑  Firmicutes ↑ Bacteroidetes↓ | *Faecalibacterium*↑ *Collinsella*↑  *Catenibacterium*↑ *Porphyromonas*↑ *Bacteroides*↑  *Phascolarctobacterium*↓ | *Catenibacterium* sp.↑  *Collinsella aerofaciens*↑  [*Bifidobacterium*](https://www.nature.com/articles/s43587-021-00129-0) adolescentis↑  *Faecalibacterium prausnitzii*↑  *Parabacteroides distasonis*↓ | Richness index↑  observed species↑ Shannon index↑ | *P*>0.05 | *Collinsella, Collinsella aerofaciens, Bifidobacterium adolescentis* were significantly positively correlated with triglycerides and significantly negatively correlated with HDL. *Collinsella, Faecalibacterium prausnitzii, Faecalibacterium* were significantly positively correlated with BMI. In addition, *Parabacteroides distasonis* was significantly positively associated with LDL |
| Zhao Y et al. 2019 | China | 58 | 9-17 | 25 obese NAFLD  18 obese non-NAFLD children 15 healthy children | Case-control study | Metagenomic shotgun sequencing | Proteobacteria↑ Firmicutes ↔ Bacteroidetes↓ | *Phascolarctobacterium* ↑ *Klebsiella*↑ *Kluyvera*↑  *Lactobacillus*↓,*Oscillibacter*↓  *Ruminiclostridium*↓  *Helicobacter*↓,*Alistipes*↓  *Paraprevotella*↓ | *Phascolarctobacterium succinatutens*↑  *Klebsiella pneumoniae*↑  *Kluyvera ascorbata*↑ *Parabacteroides johnsonii*↓  *Helicobacter pylori*↓ | Shannon index↔ | NA | NA |
| Michail S et al. 2015 | USA | 50 | 13 | 26 Healthy  13 NAFLD  11Obese no evidence of NAFLD | Cross-sectional study | Metagenomic shotgun sequencing | Actinobacteria↑ Fusobacteria↑  Alphaproteobacteria↓ Firmicutes↔ Bacteroidete↔ | *Prevotella*↑  *Epsilonproteobacteria*↑  *Gammaproteobacteria*↑  *Erysipelotrichi* ↓ *Clostridia*↓ | NA | NA | NA | NA |
| Pan X et al. 2021 | China | 75 | 7-16 | 25 Nonalcoholic fatty liver  25 Nonalcoholic steatohepatitis25 Obese and without NAFLD | Case-control study | 16S rRNA | Verrucomicrobia↓ | *Acidaminococcus*↑ *Peptoniphilus*↑ *Novosphingobium*↑*Akkermansia* ↓  *Haemophilus* ↓  *Faecalibacterium* ↓  *Agathobacter* ↓ | *Clostridium spp.* ↓ *Coprococcus comes* ↓  *Roseburia inulinivorans*↓ *Bifidobacterium adolescentis*↓ | Shannon Index↓ | *P*<0.05 | NA |
| Testerman T et al. 2022 | USA | 36 | 12 | 18non-NAFLD  18 NAFLD | Cross-sectional study | Metagenomic shotgun sequencing | Actinobacteria ↔  Firmicutes↔, Verrucomicrobia↔ Bacteroidetes↓ | *Bacteroides* ↓ *Flavonifractor* ↓ | *Actinomyces sp.* ↑  *Fusicatenibacter saccharivorans* ↑  *Romboutsia ilealis*↑  *Bacteroides thetaiotaomicron*↓ | Shannon Index↔ | *P*<0.05 | NA |
| Orsso CE et al. 2021 | Canada | 21 | 10-16 | 7 HOMA-IR Lowest Tertile  7 HOMA-IR Middle Tertile  7 HOMA-IR Highest Tertile | Cross-sectional study | Metagenomic shotgun sequencing | Bacteroidetes↔, Firmicutes↔  Actinobacteria↔,Proteobacteria↔ |  | *Bacteroides caccae*↔  *Alistipes putredinis* ↔  *Bacteroides uniformis*↔  *Faecalibacterium prausnitzii*↔ *Bacteroides vulgatus*↔  *Veillonella parvula*↓ *Dialister invisus*↓ *Haemophilus parainfluenzae*↓ | Richness index↓ | NA | NA |
| Alcazar M et al. 2022 | Spain | 191 | 8-14 | 106Metabolically healthy obesity  85Metabolically unhealthy obesity | Cross-sectional study | 16S rRNA | Bacteroidota↑ Proteobacteria↔  Actinobacteriota↔Firmicutes↓  verrucomicrobiota↓ | *Bacteroides*↑*Akkermansia*↓  *Christensenellaceae R7-group*↓ |  | Chao1index↓  Simpson index↓ | *P*>0.05 | The metabolic risk score was positively associated with Bacteroidota and Proteobacteria phyla and inversely associated with Firmicutes ,Christensenellaceae  R7-group and *Akkermansia* genus. Christensenellaceae R-7 group also was associated inversely with the triglyceride levels BMI. |
| Luoto R et al. 2011 | Finland | 30 | 10 | 15 Overweight children  15 Normal-weight children | Nested case-cohort study | FISH |  | *Bacteroides*↑,*Bifidobacterial*↓ |  | NA | NA | NA |
| Zhu L et al. 2013 | USA | 63 | 12-13 | 22NASH patients  25 Obese patients  16healthy controls | Case-control study | 16S rRNA | Bacteroidetes↑, Proteobacteria↑  Firmicutes↓, Actinobacteria↓ | *Prevotella*↑, *Peptoniphilus*↑  *Escherichia*↑,*Campylobacter*↔  *Porphyromonas*, *Clostridium*↔  *Faecalibacterium*↔  *Ruminococcus*↔*Bifidobacterium*↓,*Oscillospira*↓  *Alistipes*↓,*Blautia*↓,*Coprococcus*↓, *Eubacterium*↓, *Roseburia*↓ |  | Phylogenetic diverstiy↔  Chao1index↓ | *P*<0.05 | NA |
| Nemchenko U M et al. 2021 | USA | 40 | 11-16 | 22 Control group  18 Obese adolescents | Case-control study | 16S rRNA  PCR | Actinobacteria↓ | NA | [*Bifidobacterium*](https://www.nature.com/articles/s41390-023-02716-w) *infantis↑*  [*Bifidobacterium*](https://www.nature.com/articles/s41390-023-02716-w) *bifidum↔*  [*Bifidobacterium*](https://www.nature.com/articles/s41390-023-02716-w) *adolescentis↔*[*Bifidobacterium*](https://www.nature.com/articles/s41390-023-02716-w) *longum↓* [*Bifidobacterium*](https://www.nature.com/articles/s41390-023-02716-w) *catenulatum↓* | NA | NA | NA |
| Hu HJ et al. 2015 | Korean | 134 | 13-16 | 67obese 67normal | Case-control study | 16S rRNA | Firmicutes/Bacteroidetes↔  Bacteroidetes↔  Firmicutes↔  Proteobacteria↔ | *Prevotella↑Dialister↔*  *Parabacteroides↔*  *Sutterella↔Bacteroides↓*  *Alistipes↓*  *Faecalibacterium↓*  *Oscillibacter↓* |  | Shannon Index↔ | *P*>0.05 | *Bacteroides* was significantly and negatively associated with BMI z-score.*Prevotella* had the opposite result.*Alistipes* was negatively correlated with BMI.TG, Tchol, and hs-crp showed a negative correlation with the proportion of *Bacteroides*,while HDLc showed a positive correlation.By contrast, TG and hs-crp showed a positive correlation with the *Prevotella* population. The *Oscillibacter* population showed a negative correlation with hs-crp. |
| Da Silva CC et al. 2020 | West Indies | 51 | 6-14 | 21obesity/overweigh 30normal | Cross-sectional study | 16S rRNA | Firmicutes↑, Bacteroidetes↓  Actinobacteria↔Proteobacteria↔ | *Lactobacillus↑ Bifidobacterium↓*  *Blautia*↔*Collinsella*↔*Faecalibacterium*↔ | NA | Phylogenetic diverstiy↓ | NA | NA |
| SM Murga-Garrido et al. 2020 | Mexico | 45 | 6-12 | 26 normal weight  19 overweight and obese | Cross-sectional study | Metagenomic shotgun sequencing | NA | *Faecalibacteriums**↑*  *Monogloguss↑*  *Dialisters↑*  *Clostridialess↑ Intestinibacter↓* | *Eubacterium sp.↑*  *Bifidobacterium psudocatenulatum↑*  *Monoglobus pectinilyticus↑*  *Faecalibacterium prausnitzii↑ Bacteroides uniformis↓*  *Intestinibacter bartlettii↓*  *Bacteroides intestinalis↓*  *Clostridium citroniae↓* | Shannon Index↓ Inversed simpson Index↓ | *P*<0.05 | NA |
| Peng,Ye et al. 2020 | Canada | 50 | 3-17 | 25 Prader–Willi syndrome  25Controls | Cross-sectional study | 16S rRNA | NA | *Bifidobacterium↑ , Blautia↑*  *Dialister↑ , Coprococcus↑*  *Neisseria↑ ,Turicibacter ↑*  *Prevotella↑,Alistipes↓*  *Oscillospira↓* | NA | Shannon index↔Chao1 index↔Simpson index↔ | NA | NA |
| Yuan, Xin et al. 2023 | China | 89 | 5-15 | 29normalweight  27mild obesity  33severe obesity | Cross-sectional study | 16S rRNA | NA | *Rothia↑ Anaerotruncus↓*  *Ruminococcus↓*  *Lactonifactor↓*  *Oscillospira↓,Alistipes↓*  *Bacteroides↓* | *Rothia mucilaginosa↑*  *Bifidobacterium longum↑Clostridium hathewayi↓*  *Alistipes putredinis↓ Bacteroides eggerthii↓*  *Bacteroides acidifaciens↓ Bacteroides uniformis↓*  *Lactonifactor longoviformis↓* | Shannon index↓  chao 1 index↔Simpson index↔  Observed species↔  Ace index↔ | *P*<0.05 | In the mildly obese population, fasting glucose was positively and negatively correlated with *A. indistinctus, A. putredinis Ruminococcus*; LBP was negatively correlated with *Clostridium hathewayi, Blautia producta.* In the severely obese population, oral glucose tolerance test 2 h plasma glucose was negatively correlated with Synergistetes, Pyramidobacter,*Veillonella parvula, P. piscolens,* and positively correlated with B. producta; INS and HOMA-IR were negatively correlated with *Haemophilus, H. parainfluenzae,* and LBP was negatively correlated with Actinobacteria, *Bifidobacterium, Lactobacillus, B. longum*. |
| Gao, X. F. et al. 2019 | China | 206 | 11-16 | 65normal weight  53overweight  87obese | Cross-sectional study | 16S rRNA | Actinobacteria *↑*  Verrucomicrobia*↑* | Collinsella*↑,*Clostridium*↑*  Rhodococcus*↑,*Streptococcus*↑*  Erwinia*↑Actinomyces*↔ | NA | Shannon index↔Simpson index↔ | NA | NA |
| Gao, X. L. et al. 2018 | China | 77 | Average age=6.4 | 39 obesity  38 control | Case-control study | 16S rRNA | Bacteroidetes↑Actinobacteria↓  Proteobacteria↓  Firmicutes↓  Verrucomicrobia↓ | NA | *Bacteroides plebeius↑*  *Parasutterella excrementihominis↑*  *Parabacteroides distasonis↑*  *Bilophila wadsworthia↑*  *Clostridium symbiosum↑*  *Megamonas funiformis↑*  *Allisonella histaminiformans↑*  *Prevotella stercorea↑*  *Oxalobacter formigenes↑Faecalibacterium prausnitzii↓*  *Bacteroides thetaiotaomicron↓*  *Blautia wexlerae↓*  *Bacteroides fragilis↓*  *Blautia luti↓*  *Anaerostipes butyraticus↓*  *Streptococcus salivarius↓*  *Bacteroides ovatus↓*  *Clostridium leptum↓*  *Erysipelatoclostridium ramosum↓*  *Lactonifactor longoviformis↓*  *Solobacterium moorei↓ Akkermansia muciniphila↓*  *Enterococcus cecorum↓*  *Clostridium sporosphaeroides↓*  *Anaerostipes caccae↓*  *Streptococcus constellatus↓*  *Eubacterium sulci↓*  *Gordonibacter pamelaeae↓*  *Alloprevotella rava↓* | Observed species↑Chao1 index↑ ACE index↑Shannon index↑ Simpson index↓ | *P*<0.05 | NA |
| Gruneck L et al. 2022 | Thailand | 127 | 5-15 | 1 severe thinness  5 thinness  83 normal weight  20 overweight  18 obese | Cross-sectional study | qPCR | Bacteroidetes↔ Firmicutes↓ | *Prevotella*↔  *Bacteroides*↔  *Akkermansia*↔  *Bifidobacterium*↔*Lactobacillus*↔  *Ruminococcus**↓* | NA | NA | NA | NA |
| Monga Kravetz A et al. 2020 | USA | 73 | average age=13.1 | 29 hepatic fat fraction <5.5%  44 hepatic fat fraction ≥5.5% | Cross-sectional study | 16S rRNA | Firmicutes/Bacteroidetes↑ Bacteroidetes ↓ | *Prevotella↓,Gemmiger**↓*  *Oscillospira↓* | NA | Shannon indexe↓ | NA | NA |
| Wei J et al. 2023 | China | 54 | 10-18 | 23metabolic syndrome  31control | Case-control study | 16S rRNA | Firmicutes/Bacteroidetes↑ | *Dialister↑,Blautia*↑  *Anaerostipes*↑  *Lachnoclostridium*↑, *Parabacteroides↓*  *Bifidobacterium↓*  *Subdoligranulum↓*  *Bacteroides↓* | NA | Chao1 index↔  ACE Index↔  Shannon Index↔  Simpson Index↔  Observed species↔ | *P*>0.05 | NA |
| Jiang L F et al. 2022 | China | 123 | average age=9.91 | 63 obesity  63 control | Case-control study | 16S rRNA | NA | *Prevotella*↑  *Coprococcus-3*↑  *Ruminococcus*↑  *Agathobacter*↑  *Collinsella*↑  *Sarcina*↑ | *Akkermansia muciniphila↓* | Chao1Index  ↓  ACE Index↓  Shannon  Index↔  Simpson  Index↔ | *P*>0.05 | NA |
| Ting Ma et al. 2023 | China | 60 | 3-5 | 30 obesity  30 control | Case-control study | 16S rRNA | Firmicutes/Bacteroidetes↑ | *Faecalibacterium*↑  *Tyzzerella*↑  *Klebsiella*↑ | NA | Chao1 Index↔, observed species↔Shannon Index↔ | *P*>0.05 | NA |
| Dongyu Zhang et al. 2019 | China | 61 | 3-6 | obesity (n=30)  control(n=31) | Cross-sectional study | qRT-PCR | NA | *Lactobacillus*↑, *Escherichia*↔*Bifidobacterium*↔, *Clostridium↓* | NA | NA | NA | Lactobacillus showed significant positive correlation with BMI in children, while Clostridium and showed significant negative correlation with BMI in children. |
| Chunfeng Tian et al. 2023 | China | 30 | 7-13 | obesity (n=15)  control(n=15) | Case-control study | 16S rRNA | Actinobacteria↑, Proteobacteria↑, Firmicutes/Bacteroides↑, Firmicutes*↓,* Bacteroidetes*↓* | *Ruminococcus*↑, *Pseudobutyrivibrio*↑, *Escherichia-Shigella*↑ *Bacteroides**↓, Faecalibacterium↓, Alistipes↓, Blautia↓* | NA | Chao1 index↔observed species↔Shannon index↔  Simpson index↔ | *P*>0.05 | NA |
| Shunfeng Mao et al. 2022 | China | 100 | 3-6 | obesity (n=50)  control(n=50) | Cross-sectional study | 16S rRNA | Proteobacteria↑,  Actinobacteria↔,  Bacteroidetes↔, Firmicutes↔ | *Bifidobacterium*↔, *Bacteroides*↔, *Lactobacillus*↔ | *Shigella coli*↔ | NA | NA | NA |
| Jing Li et al. 2015 | China | 63 | 4-6 | obesity (n=29)  control(n=34) | Cross-sectional study | 16S rRNA | Proteobacteria↑,  Actinobacteria↔,  Bacteroidetes↔, Firmicutes↔ | *Anaerococcus*↑, *Cloacibacillus*↑, *Mitsuokella*↑, *Mesorhizobium*↑, *Bifidobacterium*↔, *Bacteroides*↔, *Lactobacillus*↔, *Butyrivibrio↓*, *Mobilu-ncus↓*, *hydrogenophaga↓*, *Lysobacter↓*, *Actinomyces↓*, *Leptotrichia↓* | *Shigella coli*↔ | NA | NA | NA |
| Hou YP et al. 2017 | China | 143 | 3-18 | obesity (n=87)  control(n=56) | Case-control study | 16S rRNA | Firmicutes↑,Proteobacteria↔Bacteroidetes*↓,* Verrucomicrobia*↓* | *Enterococcus*↑*,  Blautia*↑*, Sutterella*↑*, Klebsiella*↑*, Collinsella*↑,*Bacteroides↓,*  *Parabacteroides↓,  Anaerotruncus↓,  Coprobacillus↓* | NA | ↔alpha diversity | *P*<0.05 | NA |

**Abbreviations:** NAFLD , Nonalcoholic Fatty Liver Disease; NASH, Non-alcoholic steatohepatitis ; IS , insulin sensitive ; IS, insulin resistance.

**Supplemental Table 2:** Risk of bias assessment for included case-control studies.

| **References** | **Were the groups comparable other than the presence of disease in cases or the absence of disease in controls?** | **Were cases and controls matched appropriately?** | **Were the same criteria used for identification of cases and controls?** | **Was exposure measured in a standard, valid and reliable way?** | **Was exposure measured in the same way for cases and controls?** | **Were confounding factors identified?** | **Were strategies to deal with confounding factors stated?** | **Were outcomes assessed in a standard, valid and reliable way for cases and controls?** | **Was the exposure period of interest long enough to be meaningful?** | **Was appropriate statistical analysis used?** |
| --- | --- | --- | --- | --- | --- | --- | --- | --- | --- | --- |
| Borgo F et al.2017 | Low risk | Low risk | Low risk | Low risk | Low risk | Low risk | Low risk | Low risk | Low risk | Low risk |
| López-Contreras BE et al.2018 | Low risk | Low risk | Low risk | Low risk | Low risk | Low risk | Low risk | Low risk | Low risk | Low risk |
| Xu P et al.2012 | Low risk | Low risk | Low risk | Low risk | Low risk | High risk | High risk | Low risk | Unsure | Low risk |
| Del Chierico F et al.2018 | Low risk | Low risk | Low risk | Low risk | Low risk | Low risk | Low risk | Low risk | Low risk | Low risk |
| Grigorova EV et al.2021 | Low risk | Low risk | Low risk | Low risk | Low risk | Low risk | Low risk | Low risk | Low risk | Low risk |
| Abdallah Ismail N et al. 2011 | Low risk | Low risk | Low risk | Low risk | Low risk | Low risk | High risk | Low risk | Low risk | Low risk |
| Karlsson CL et al. 2012 | Low risk | Low risk | Low risk | Low risk | Low risk | Low risk | High risk | High risk | Low risk | Low risk |
| Chen X et al. 2020 | Low risk | Low risk | Low risk | Low risk | Low risk | Low risk | Low risk | Low risk | Low risk | Low risk |
| Balamurugan R et al. 2010 | Low risk | Low risk | Low risk | Low risk | Low risk | High risk | High risk | Low risk | High risk | Low risk |
| Gallardo-Becerra L et al. 2020 | Low risk | Low risk | Low risk | Low risk | Low risk | Unsure | Unsure | High risk | Low risk | Low risk |
| Zhao Y et al. 2019 | Low risk | Low risk | Low risk | Low risk | Low risk | Low risk | Low risk | Low risk | Low risk | Low risk |
| Pan X et al. 2021 | Low risk | Low risk | Low risk | Low risk | Low risk | Unsure | Unsure | High risk | Low risk | Low risk |
| Luoto R et al. 2011 | Low risk | Low risk | Low risk | Low risk | Low risk | High risk | High risk | Low risk | Low risk | Low risk |
| Zhu L et al. 2013 | Low risk | Low risk | Low risk | Low risk | Low risk | Unsure | Unsure | Low risk | Unsure | Low risk |
| Nemchenko U M et al. 2021 | Low risk | Low risk | Low risk | Low risk | Low risk | Low risk | Low risk | Low risk | Low risk | Low risk |
| Hu HJ et al. 2015 | Low risk | Low risk | Low risk | Low risk | Low risk | Low risk | Low risk | Low risk | High risk | Low risk |
| Gao, X. L. et al. 2018 | Low risk | Low risk | Low risk | Low risk | Low risk | High risk | High risk | Low risk | Unsure | Low risk |
| Wei J et al. 2023 | Low risk | Low risk | Low risk | Low risk | Low risk | Low risk | Low risk | Low risk | Unsure | Low risk |
| Jiang L F et al. 2022 | Low risk | Low risk | Low risk | Low risk | Low risk | Low risk | Low risk | Low risk | Low risk | Low risk |
| Ting Ma et al. 2023 | Low risk | Low risk | Low risk | Low risk | Low risk | Low risk | Low risk | Low risk | Low risk | Low risk |
| Chunfeng Tian et al. 2023 | Low risk | Low risk | Low risk | Low risk | Low risk | High risk | High risk | Low risk | Unsure | Low risk |
| Hou YP et al. 2017 | Low risk | Unsure | Low risk | Low risk | Low risk | High risk | High risk | Low risk | Unsure | Low risk |

**Supplemental Table 3:** Risk of bias assessment for included cross-sectional studies.

| **References** | **Were the criteria for inclusion in the sample clearly defined?** | **Were the study subjects and the setting described in detail?** | **Was the exposure measured in a valid and reliable way?** | | **Were objective, standard criteria used for measurement of the condition?** | | **Were confounding factors identified?** | | **Were strategies to deal with confounding factors stated?** | | **Were the outcomes measured in a valid and reliable way?** | | **Was appropriate statistical analysis used?** |
| --- | --- | --- | --- | --- | --- | --- | --- | --- | --- | --- | --- | --- | --- |
| Vazquez-Moreno M et al.2021 | Yes | Yes | Yes | Yes | | No | | No | | Yes | | Yes | |
| Gyarmati P et al.2021 | Yes | Yes | Yes | Yes | | No | | No | | Yes | | Yes | |
| Benítez-Páez A et al.2020 | Yes | Yes | Yes | Yes | | Yes | | Yes | | Yes | | Yes | |
| Shin S et al. 2020 | Yes | Yes | Yes | Yes | | Yes | | Yes | | Yes | | Yes | |
| Barczyńska R et al.2018 | Yes | Yes | Unsure | No | | No | | No | | Yes | | Yes | |
| Méndez-Salazar EO et al. 2018 | Yes | Yes | Yes | Yes | | Unsure | | Unsure | | Yes | | Yes | |
| Bervoets L et al.2013 | Yes | Yes | Yes | No | | Yes | | Yes | | Yes | | Yes | |
| Visuthranukul C et al.2022 | Yes | Yes | Yes | Yes | | Yes | | Yes | | Yes | | Yes | |
| Maya-Lucas O et al.2019 | Yes | Yes | Unsure | Yes | | Yes | | Yes | | Yes | | Yes | |
| Orbe-Orihuela YC et al.2018 | Yes | Unsure | Yes | Yes | | No | | No | | Yes | | Yes | |
| Balakrishnan B et al.2021 | Yes | Unsure | Yes | No | | Yes | | Yes | | Yes | | Yes | |
| Del Chierico F et al.2021 | Yes | Yes | Yes | Yes | | Yes | | Yes | | Yes | | Yes | |
| Nirmalkar K et al.2018 | Yes | Yes | Yes | Yes | | Yes | | Yes | | Yes | | Yes | |
| Yuan X et al.2021 | Yes | Yes | Yes | Yes | | Yes | | Yes | | Yes | | Yes | |
| Ferrer M et al.2013 | Yes | Unsure | Yes | Yes | | No | | No | | Yes | | Yes | |
| Goffredo M et al.2016 | Yes | No | Yes | Yes | | Yes | | Yes | | Yes | | Yes | |
| lgnacio A et al. 2016 | Yes | Yes | Yes | Yes | | Yes | | Yes | | Yes | | Yes | |
| Jaimes JD et al. 2021 | Yes | Yes | Yes | Yes | | Unsure | | Unsure | | Yes | | Yes | |
| Murugesan S et al. 2015 | Yes | Yes | Yes | Yes | | Unsure | | Unsure | | Yes | | Yes | |
| Chen X et al. 2021 | Yes | Yes | Yes | Yes | | No | | No | | Yes | | Yes | |
| Pierri L et al. 2018 | Yes | Yes | Yes | Yes | | Unsure | | Unsure | | Yes | | Yes | |
| Yuan X et al. 2021 | Yes | Yes | Yes | Yes | | No | | No | | Yes | | Yes | |
| Wei Y et al. 2021 | Yes | Yes | Yes | Yes | | Yes | | Yes | | Yes | | Yes | |
| Mousavi S H et al. 2008 | Yes | Yes | Yes | Yes | | Unsure | | Unsure | | Yes | | Yes | |
| Michail S et al. 2015 | Yes | Yes | Yes | Yes | | Yes | | Yes | | Yes | | Yes | |
| Testerman T et al. 2022 | Yes | Yes | Yes | Yes | | Yes | | Yes | | Yes | | Yes | |
| Orsso CE et al. 2021 | Yes | Yes | Yes | Yes | | Yes | | Yes | | Yes | | Yes | |
| Alcazar M et al. 2022 | Yes | Yes | Yes | Yes | | Yes | | Yes | | Yes | | Yes | |
| Da Silva CC et al. 2020 | Yes | Yes | Yes | Yes | | Unsure | | Unsure | | Yes | | Yes | |
| SM Murga-Garrido et al. 2020 | Yes | Yes | Yes | Yes | | Yes | | Yes | | Yes | | Yes | |
| Peng,Ye et al. 2020 | Yes | Yes | Yes | Yes | | Yes | | Yes | | Yes | | Yes | |
| Yuan, Xin et al. 2023 | Yes | Yes | Yes | Yes | | Yes | | Yes | | Yes | | Yes | |
| Gao, X. F. et al. 2019 | Yes | Yes | Yes | Yes | | Unsure | | Unsure | | Yes | | Yes | |
| Gruneck L et al. 2022 | Yes | Yes | Yes | Yes | | No | | No | | Yes | | Yes | |
| Monga Kravetz A et al. 2020 | Yes | Yes | Yes | Yes | | Yes | | Yes | | Yes | | Yes | |
| Jing Li et al. 2015 | Yes | Yes | Yes | Yes | | No | | No | | Yes | | Yes | |
| Dongyu Zhang et al. 2019 | Yes | Yes | Yes | Yes | | No | | No | | Yes | | Yes | |
| Shunfeng Mao et al. 2022 | Yes | Yes | Yes | Yes | | No | | No | | Yes | | Unsure | |

**Supplemental Table 4:** Risk of bias assessment for included cohort studies.

| **References** | **Were the two groups similar and recruited from the same population?** | **Were the exposures measured similarly to assign people to both exposed an unexposed groups?** | **Was the exposure measured in a valid and reliable way?** | **Were confounding factors identified?** | **Were strategies to deal with confounding factors stated?** | **Were the groups/participants free of the outcome at the start of the study (or at the moment of exposure)?** | **Were the outcomes measured in a valid and reliable way?** | **Was the follow up time reported and sufficient to be long enough for outcomes to occur?** | **Was follow up complete, and if not, were the reasons to loss to follow up described and explored?** | **Were strategies to address incomplete follow up utilized?** | **Was appropriate statistical analysis used?** |
| --- | --- | --- | --- | --- | --- | --- | --- | --- | --- | --- | --- |
| Karvonen AM et al.2019 | Yes | Yes | Yes | Yes | Yes | unsure | Yes | No | Yes | Unsure | Yes |
| Riva A et al.2017 | Yes | Yes | Yes | Unsure | Unsure | unsure | Yes | Yes | Yes | No | Yes |
| Cho KY et al. 2018 | Yes | Yes | Yes | Unsure | Unsure | Yes | Yes | Unsure | Unsure | No | Yes |

**Supplemental documents**

1. **Full-text articles excluded, with reasons (n=42)**

1.	Strati F, Di Paola M, Stefanini I, Albanese D, Rizzetto L, Lionetti P, Calabrò A, Jousson O, Donati C, Cavalieri D, De Filippo C. Age and Gender Affect the Composition of Fungal Population of the Human Gastrointestinal Tract. Front Microbiol. 2016;7:1227. doi: 10.3389/fmicb.2016.01227. PMID: 27536299.

2.	Du W, Liu L, Ma Y, Zhu Q, Jia R, Han Y, Wu Z, Yan X, Ailizire A, Zhang W. Analysis of the gut microbiome in obese native Tibetan children living at different altitudes: A case-control study. Front Public Health. 2022;10:963202. doi: 10.3389/fpubh.2022.963202. PMID: 36504960.

3.	Das B, Ghosh TS, Kedia S, Rampal R, Saxena S, Bag S, Mitra R, Dayal M, Mehta O, Surendranath A, Travis SPL, Tripathi P, Nair GB, Ahuja V. Analysis of the Gut Microbiome of Rural and Urban Healthy Indians Living in Sea Level and High Altitude Areas. Sci Rep. 2018;8:10104. doi: 10.1038/s41598-018-28550-3. PMID: 29973712.

4.	Shaw AG, Black N, Rushd A, Sim K, Randell P, Kroll JS, Epstein J. Assessing the Colonic Microbiota in Children: Effects of Sample Site and Bowel Preparation. J Pediatr Gastroenterol Nutr. 2017;64:230-7. doi: 10.1097/mpg.0000000000001233. PMID: 27070657.

5.	Singhal V, Youssef S, Bredella M, Misra M. Associations Of Bile Acids And Gut Microbiome With Insulin Resistance In Adolescents With Obesity. OBESITY: WILEY 111 RIVER ST, HOBOKEN 07030-5774, NJ USA, 2020:56-.

6.	Santarossa S, Sitarik AR, Johnson CC, Li J, Lynch SV, Ownby DR, Ramirez A, Yong GL, Cassidy-Bushrow AE. Associations of physical activity with gut microbiota in pre-adolescent children. Phys Act Nutr. 2021;25:24-37. doi: 10.20463/pan.2021.0023. PMID: 35152621.

7.	Chen SS, Liao XM, Wei QZ, Zhou YY, Su MY, Hu Y, Song YY, Zhang ZQ, Liang JJ. Associations of the Gut Microbiota Composition and Fecal Short-Chain Fatty Acids with Leukocyte Telomere Length in Children Aged 6 to 9 Years in Guangzhou, China: A Cross-sectional Study. J Nutr. 2022;152:1549-59. doi: 10.1093/jn/nxac063. PMID: 35278080.

8.	Duan M, Wang Y, Zhang Q, Zou R, Guo M, Zheng H. Characteristics of gut microbiota in people with obesity. PLoS One. 2021;16:e0255446. doi: 10.1371/journal.pone.0255446. PMID: 34375351.

9.	Yasir M, Angelakis E, Bibi F, Azhar E, Bachar D, Lagier J, Gaborit B, Hassan A, Jiman-Fatani A, Alshali K. Comparison of the gut microbiota of people in France and Saudi Arabia. Nutr Diabetes. 2015;5:e153-e. doi: 10.1038/nutd.2015.3. PMID: 25915742.

10.	Lee S, Sung J, Lee J, Ko G. Comparison of the gut microbiotas of healthy adult twins living in South Korea and the United States. Applied and environmental microbiology. 2011;77:7433-7. doi: 10.1128/AEM.05490-11. PMID:21873488.

11.	de Meij TG, Budding AE, de Groot EF, Jansen FM, Kneepkens CF, Benninga MA, Penders J, van Bodegraven AA, Savelkoul PH. Composition and stability of intestinal microbiota of healthy children within a Dutch population. The FASEB Journal. 2016;30:1512-22. doi: 10.1096/fj.15-278622. PMID:26655704.

12.	Bai J, Hu Y, Bruner D. Composition of gut microbiota and its association with body mass index and lifestyle factors in a cohort of 7–18 years old children from the American Gut Project. Pediatric obesity. 2019;14:e12480. doi:10.1111/ijpo.12480. PMID: 30417607.

13.	Khan M, Quince C, Ijaz U, Loman N, Calus S, Quick J, Haig S, Shaikh M, Edwards C, Gerasimidis K. A detailed analysis of the gut microbial diversity and metabolic activity in children with obesity of different aetiology and lean controls. Proceedings of the Nutrition Society. 2015;74:E75. doi: 10.1017/S0029665115000907.

14.	Agans R, Rigsbee L, Kenche H, Michail S, Khamis HJ, Paliy O. Distal gut microbiota of adolescent children is different from that of adults. FEMS microbiology ecology. 2011;77:404-12. doi: 10.1111/j.1574-6941.2011.01120.x. PMID:21539582

15.	Lin A, Bik EM, Costello EK, Dethlefsen L, Haque R, Relman DA, Singh U. Distinct distal gut microbiome diversity and composition in healthy children from Bangladesh and the United States. PloS one. 2013;8:e53838. doi: 10.1007/s00203-015-1089-0. PMID: 25644242

16.	La-Ongkham O, Nakphaichit M, Leelavatcharamas V, Keawsompong S, Nitisinprasert S. Distinct gut microbiota of healthy children from two different geographic regions of Thailand. Arch Microbiol. 2015;197:561-73. doi: 10.1007/s00203-015-1089-0. PMID: 25644242.

17.	Kim M-S, Park E-J, Roh SW, Bae J-W. Diversity and abundance of single-stranded DNA viruses in human feces. Applied and environmental microbiology. 2011;77:8062-70. doi: 10.1128/AEM.06331-11. PMID: 21948823.

18.	Nakayama J, Watanabe K, Jiang J, Matsuda K, Chao S-H, Haryono P, La-Ongkham O, Sarwoko M-A, Sujaya IN, Zhao L. Diversity in gut bacterial community of school-age children in Asia. Scientific reports. 2015;5:8397. doi: 10.1038/srep08397.

PMID: 25703686.

19.	Radjabzadeh D, Boer CG, Beth SA, van der Wal P, Kiefte-De Jong JC, Jansen MA, Konstantinov SR, Peppelenbosch MP, Hays JP, Jaddoe VW. Diversity, compositional and functional differences between gut microbiota of children and adults. Scientific reports. 2020;10:1040. doi: 10.1038/s41598-020-57734-z. PMID: 31974429.

20.	Schei K, Simpson MR, Avershina E, Rudi K, Øien T, Júlíusson PB, Underhill D, Salamati S, Ødegård RA. Early Gut Fungal and Bacterial Microbiota and Childhood Growth. Front Pediatr. 2020;8:572538. doi: 10.3389/fped.2020.572538. PMID: 33240830.

21.	Mello CS, Rodrigues M, Filho HBA, Melli L, Tahan S, Pignatari ACC, de Morais MB. Fecal microbiota analysis of children with small intestinal bacterial overgrowth among residents of an urban slum in Brazil. J Pediatr (Rio J). 2018;94:483-90. doi: 10.1016/j.jped.2017.09.003. PMID: 29049893.

22.	Borges FM, de Paula TO, Sarmiento MR, de Oliveira MG, Pereira ML, Toledo IV, Nascimento TC, Ferreira-Machado AB, Silva VL, Diniz CG. Fungal diversity of human gut microbiota among eutrophic, overweight, and obese individuals based on aerobic culture-dependent approach. Current microbiology. 2018;75:726-35. doi: 10.1007/s00284-018-1438-8. PMID: 29368026.

23.	Zuo H-J, Xie Z-M, Zhang W-W, Li Y-R, Wang W, Ding X-B, Pei X-F. Gut bacteria alteration in obese people and its relationship with gene polymorphism. World journal of gastroenterology: WJG. 2011;17:1076. doi: 10.3748/wjg.v17.i8.1076.

PMID: 21448362

24.	Aguilar T, Nava GM, Olvera-Ramírez AM, Ronquillo D, Camacho M, Zavala GA, Caamaño MC, Acevedo-Whitehouse K, Rosado JL, García OP. Gut bacterial families are associated with body composition and metabolic risk markers in school-aged children in rural Mexico. Child Obes. 2020;16:358-66. doi:10.1089/chi.2019.0312. PMID: 32429742 .

25.	Mello CS, Carmo-Rodrigues MS, Humberto Filho B, Melli LC, Tahan S, Pignatari AC, de Morais MB. Gut microbiota differences in children from distinct socioeconomic levels living in the same urban area in Brazil. Journal of Pediatric Gastroenterology and Nutrition. 2016;63:460-5. doi: 10.1097/MPG.0000000000001186. PMID: 26982745

26.	Palacios‐González B, Mendez E, Ortiz‐Lopez MG, de los Angeles Granados‐Silvestre M, Menjívar M. Intestinal dysbiosis in obese and undernourished Mexican school‐age children. The FASEB Journal. 2016;30:1166.8-.8. doi: 10.1096/fasebj.30.1_supplement.1166.8.

27.	Bezek K, Petelin A, Pražnikar J, Nova E, Redondo N, Marcos A, Jenko Pražnikar Z. Obesity Measures and Dietary Parameters as Predictors of Gut Microbiota Phyla in Healthy Individuals. Nutrients. 2020;12. doi: 10.3390/nu12092695. PMID: 32899326.

28.	Singhal V, Youssef S, Harshman S, Stanford F, Slattery M, Lawson E, Eddy K, Bredella M, Misra M. Obesity related differences in gut microbiome in adolescents may be modified by sex. Horm Res Paediatr: KARGER ALLSCHWILERSTRASSE 10, CH-4009 BASEL, SWITZERLAND, 2020:22-3.

29.	Nemchenko UM, Grigorova EV, Pogodina AV, Belkova NL, Voropaeva NM, Romanitsa AI, Grigoryeva EA, Savelkaeva MV, Rychkova LV. The relationship between the composition of the microbiota and functional intestinal disorders in obese adolescents. Klin Lab Diagn. 2022;67:106-9. doi: 10.51620/0869-2084-2022-67-2-106-109. PMID: 35192757.

30.	Gurova M, Novikova V, Khavkin A. The state of gut microbiota and clinical-metabolic features in children with overweight and obesity. Russian journal of evidence-based gastroenterology. 2018;7:4-10.

31.	Shin S, Cho KY. Altered Gut Microbiota and Shift in Bacteroidetes between Young Obese and Normal-Weight Korean Children: A Cross-Sectional Observational Study. Biomed Res Int. 2020;2020:6587136. doi: 10.1155/2020/6587136. PMID: 32908903.

32.	Stanislawski MA, Dabelea D, Wagner BD, Iszatt N, Dahl C, Sontag MK, Knight R, Lozupone CA, Eggesbø M. Gut Microbiota in the First 2 Years of Life and the Association with Body Mass Index at Age 12 in a Norwegian Birth Cohort. mBio. 2018;9. doi: 10.1128/mBio.01751-18. PMID: 30352933.

33.	Selma-Royo M, Cortés-Macías E, Sánchez G, Collado MC. 1.4.5 Gut Microbiota Development in Infants and Children. World Rev Nutr Diet. 2022;124:107-14. doi: 10.1159/000516714. PMID: 35240614.

34.	Ville A, Levine E, Zhi D, Lararia B, Wojcicki JM. Alterations in the gut microbiome at 6 months of age in obese latino infants. Journal of the American College of Nutrition. 2020;39:47-53. doi: 10.1080/07315724.2019.1606744. PMID: 31498715.

35.	Wang Y, Hoenig JD, Malin KJ, Qamar S, Petrof EO, Sun J, Antonopoulos DA, Chang EB, Claud EC. 16S rRNA gene-based analysis of fecal microbiota from preterm infants with and without necrotizing enterocolitis. The ISME journal. 2009;3:944-54. doi: 10.1038/ismej.2009.37. PMID: 19369970 .

36.	Holmes ZC, Silverman JD, Dressman HK, Wei Z, Dallow EP, Armstrong SC, Seed PC, Rawls JF, David LA. Short-chain fatty acid production by gut microbiota from children with obesity differs according to prebiotic choice and bacterial community composition. MBio. 2020;11:10.1128/mbio. 00914-20. doi: 10.1128/mBio.00914-20. PMID: 32788375.

37.	Herman DR, Pham A-MR, Lopez U, Flores GE. Childhood Obesity and the Gut Microbiome : What are the Dietary Drivers? The FASEB Journal. 2016;30:1152.12-.12. doi: 10.1096/fasebj.30.1_supplement.1152.12.

38.	Palacios-González B, Mendez E, Ortiz-Lopez MG, de los Angeles Granados-Silvestre M, Menjívar M. Intestinal dysbiosis in obese and undernourished Mexican school-age children. The FASEB Journal. 2016;30:1166.8-.8. doi: 10.1096/fasebj.30.1_supplement.1166.8.

39.	Hefley S, Howard N, Crasto C, Vasylyeva T. Microbiome Dysbiosis in Obese Adolescents: A Potential Contributor to Inflammation. The FASEB Journal. 2021;35. doi: 10.1096/fasebj.2021.35.S1.02509.

40.	Kong XJ, Liu K, Zhuang P, Tian R, Liu S, Clairmont C, Lin X, Sherman H, Zhu J, Wang Y, et al. The Effects of Limosilactobacillus reuteri LR-99 Supplementation on Body Mass Index, Social Communication, Fine Motor Function, and Gut Microbiome Composition in Individuals with Prader-Willi Syndrome: a Randomized Double-Blinded Placebo-Controlled Trial. Probiotics Antimicrob Proteins. 2021;13:1508‐20. doi: 10.1007/s12602-021-09800-9. PMID: CN-02287492.

41.	Jones RB, Alderete TL, Martin AA, Geary BA, Hwang DH, Palmer SL, Goran MI. Probiotic supplementation increases obesity with no detectable effects on liver fat or gut microbiota in obese Hispanic adolescents: a 16-week, randomized, placebo-controlled trial. Pediatric obesity. 2018;13:705‐14. doi: 10.1111/ijpo.12273. PMID: CN-01466288.

42.	Hollister EB, Foster BA, Dahdouli M, Ramirez J, Lai Z. Characterization of the Stool Microbiome in Hispanic Preschool Children by Weight Status and Time. Childhood obesity (Print). 2018;14:122‐30. doi: 10.1089/chi.2017.0122. PMID: CN-01708552.

**Not outcome of interest(n=35)**

1.	Miranda VPN, Dos Santos Amorim PR, Bastos RR, de Faria ER, de Castro Moreira ME, do Carmo Castro Franceschini S, do Carmo Gouveia Peluzio M, de Luces Fortes Ferreira CL, Priore SE. Abundance of Gut Microbiota, Concentration of Short-Chain Fatty Acids, and Inflammatory Markers Associated with Elevated Body Fat, Overweight, and Obesity in Female Adolescents. Mediators Inflamm. 2019;2019:7346863. doi: 10.1155/2019/7346863. PMID: 31933541.

2.	Sepp E, Lõivukene K, Julge K, Voor T, Mikelsaar M. The association of gut microbiota with body weight and body mass index in preschool children of Estonia. Microb Ecol Health Dis. 2013;24. doi: 10.3402/mehd.v24i0.19231. PMID: 24009544.

3.	Demirbaş F, Çaltepe G, Comba A, Abbasguliyev H, Yurttan Uyar N, Kalaycı AG. Association of obesity and non-alcoholic fatty liver disease with the fecal calprotectin level in children. Arab J Gastroenterol. 2020;21:211-5. doi: 10.1016/j.ajg.2020.09.003. PMID: 33248976.

4.	Nobili V, Putignani L, Mosca A, Del Chierico F, Vernocchi P, Alisi A, Stronati L, Cucchiara S, Toscano M, Drago L. Bifidobacteria and lactobacilli in the gut microbiome of children with non-alcoholic fatty liver disease: which strains act as health players? Arch Med Sci. 2018;14:81-7. doi: 10.5114/aoms.2016.62150. PMID: 29379536.

5.	Journey EK, Ortega-Santos CP, Bruening M, Whisner CM. Changes in Weight Status and the Intestinal Microbiota Among College Freshman, Aged 18 Years. J Adolesc Health. 2020;66:166-71. doi: 10.1016/j.jadohealth.2019.06.005. PMID: 31477511.

6.	Zhang N, Ma G. Childhood obesity in China: trends, risk factors, policies and actions. Global Health Journal. 2018;2:1-13. doi: 10.1016/S2414-6447(19)30115-0

7.	Estrada-Velasco BI, Cruz M, Garcia-Mena J, Madrid-Marina V, Burguete-García A. Childhood obesity is associated to the interaction between firmicutes and high energy food consumption. Nutricion Hospitalaria. 2014;31:1074-81. doi: 10.3305/nh.2015.31.3.8302. PMID: 25726195.

8.	Pérez-Herrera A, Cruz-López M. Childhood obesity: current situation in Mexico. Nutricion Hospitalaria. 2019;36:463-9. doi: 10.20960/nh.2116. PMID: 30866635.

9.	Nemchenko U, Savelkaeva M, Grigorova E, Ivanova E, Pogodina A, Rychkova L. Comparison of microecological intestinal indices in children with different value of body mass index. Acta biomedica scientifica. 2017;2:111-5.

10.	Śliżewska K, Włodarczyk M, Sobczak M, Barczyńska R, Kapuśniak J, Socha P, Wierzbicka-Rucińska A, Kotowska A. Comparison of the Activity of Fecal Enzymes and Concentration of SCFA in Healthy and Overweight Children. Nutrients. 2023;15:987. doi: 10.3390/nu15040987. PMID: 36839343.

11.	Payne AN, Chassard C, Banz Y, Lacroix C. The composition and metabolic activity of child gut microbiota demonstrate differential adaptation to varied nutrient loads in an in vitro model of colonic fermentation. FEMS microbiology ecology. 2012;80:608-23. doi: 10.1111/j.1574-6941.2012.01330.x. PMID: 22324938.

12.	Henderson M, Van Hulst A, Simoneau G, Barnett TA, Drapeau V, Mathieu M-È, Nicolau B, Varin T, Marette A. Correlations between Measures of Adiposity Across Childhood and Adolescence and the Intestinal Microbiota in 15-17 Year-Old Children with a Family History of Obesity: Preliminary Findings from the QUALITY Cohort. 2018.

13.	Klimenko E, Belkova N, Romanitsa A, Pogodina A, Rychkova L, Darenskaya M. Differences in gut microbiota composition and predicted metabolic functions: a pilot study of adolescents with normal weight and obesity. Bulletin of Experimental Biology and Medicine. 2022;173:628-32. doi: 10.1007/s10517-022-05601-4.

14.	Moran-Ramos S, Lopez-Contreras BE, Villarruel-Vazquez R, Ocampo-Medina E, Macias-Kauffer L, Martinez-Medina JN, Villamil-Ramirez H, León-Mimila P, Del Rio-Navarro BE, Ibarra-Gonzalez I, Vela-Amieva M, Gomez-Perez FJ, Velazquez-Cruz R, Salmeron J, Reyes-Castillo Z, Aguilar-Salinas C, Canizales-Quinteros S. Environmental and intrinsic factors shaping gut microbiota composition and diversity and its relation to metabolic health in children and early adolescents: A population-based study. Gut Microbes. 2020;11:900-17. doi: 10.1080/19490976.2020.1712985.

15.	Gomes AT, Novaes TG, Silveira KC, Souza CL, Lamounier JA, Netto MP, Capanema FD, Rocha DdS. Excess weight and factors associated in preschool of southwest of Bahia. Revista Brasileira de Saúde Materno Infantil. 2017;17:365-73. doi: 10.1590/1806-93042017000200009.

16.	Digitale J, Sié A, Coulibaly B, Ouermi L, Dah C, Tapsoba C, Bärnighausen T, Lebas E, Arzika AM, Glymour MM. Gut Bacterial Diversity and Growth among Preschool Children in Burkina Faso. The American Journal of Tropical Medicine and Hygiene. 2020;103:2568. doi: 10.4269/ajtmh.20-0059. PMID: 32996444.

17.	Van de Pol JA, Van Best N, Mbakwa CA, Thijs C, Savelkoul PH, Arts IC, Hornef MW, Mommers M, Penders J. Gut colonization by methanogenic archaea is associated with organic dairy consumption in children. Front Microbiol. 2017;8:355. doi: 10.3389/fmicb.2017.00355. PMID: 28344572 .

18.	Bikel S, López-Leal G, Cornejo-Granados F, Gallardo-Becerra L, García-López R, Sánchez F, Equihua-Medina E, Ochoa-Romo JP, López-Contreras BE, Canizales-Quinteros S. Gut dsDNA virome shows diversity and richness alterations associated with childhood obesity and metabolic syndrome. iScience. 2021;24. doi: 10.1016/j.isci.2021.102900. PMID: 34409269.

19.	Houtman TA, Eckermann HA, Smidt H, de Weerth C. Gut microbiota and BMI throughout childhood: The role of firmicutes, bacteroidetes, and short-chain fatty acid producers. Scientific Reports. 2022;12:3140. doi: 10.1038/s41598-022-07176-6. PMID: 35210542.

20.	Stanislawski MA, Lozupone CA, Wagner BD, Eggesbø M, Sontag MK, Nusbacher NM, Martinez M, Dabelea D. Gut microbiota in adolescents and the association with fatty liver: the EPOCH study. Pediatric research. 2018;84:219-27. doi: 10.1038/pr.2018.32PMID: 29538359.

21.	Scheepers L, Penders J, Mbakwa C, Thijs C, Mommers M, Arts I. The intestinal microbiota composition and weight development in children: the KOALA Birth Cohort Study. International journal of obesity. 2015;39:16-25. doi: 10.1038/ijo.2014.178. PMID: 25298274.

22.	Marinov Z, Pastucha D. Komplex metabolic changes in children with obesity. Pediatrie pro Praxi. 2012;13:12-5.

23.	Payne AN, Chassard C, Zimmermann M, Müller P, Stinca S, Lacroix C. The metabolic activity of gut microbiota in obese children is increased compared with normal-weight children and exhibits more exhaustive substrate utilization. Nutr Diabetes. 2011;1:e12. doi: 10.1038/nutd.2011.8. PMID: 23154580.

24.	Vanderhaeghen S, Lacroix C, Schwab C. Methanogen communities in stools of humans of different age and health status and co-occurrence with bacteria. FEMS Microbiol Lett. 2015;362:fnv092. doi: 10.1093/femsle/fnv092. PMID: 26025070.

25.	Kim EJ, Kim HJ. Nonalcoholic fatty liver disease in obese and nonobese pediatric patients. Korean J Pediatr. 2019;62:30-5. doi: 10.3345/kjp.2018.06786. PMID: 30304905.

26.	Rampelli S, Guenther K, Turroni S, Wolters M, Veidebaum T, Kourides Y, Molnár D, Lissner L, Benitez-Paez A, Sanz Y. Pre-obese children’s dysbiotic gut microbiome and unhealthy diets may predict the development of obesity. Commun Biol. 2018;1:222. doi: 10.1038/s42003-018-0221-5. PMID: 30534614.

27.	Moossavi S, Azad MB. Quantifying and Interpreting the Association between Early-Life Gut Microbiota Composition and Childhood Obesity. mBio. 2019;10. doi: 10.1128/mBio.02787-18. PMID: 30755514.

28.	Belei O, Olariu L, Dobrescu A, Marcovici T, Marginean O. The relationship between non-alcoholic fatty liver disease and small intestinal bacterial overgrowth among overweight and obese children and adolescents. J Pediatr Endocrinol Metab. 2017;30:1161-8. doi: 10.1515/jpem-2017-0252. PMID: 28988228.

29.	Bervoets L, Van Hoorenbeeck K, Kortleven I, Van Noten C, Hens N, Vael C, Goossens H, Desager K, Vankerckhoven V. Relationship between the gut microbiota, diet, physical activity and obesity in children. Obesity Facts. 2012;5:41. doi: 10.1159/000188329.

30.	Shestopalov A, Shatova O, Gaponov A, Moskaleva N, Appolonova S, Tutelyan A, Makarov V, Yudin S, Rumyantsev S. The study of tryptophan metabolite concentrations in blood serum and fecal extracts from obese children. Biomeditsinskaya khimiya. 2020;66:494-501. doi: 10.18097/PBMC20206606494. PMID: 33372908 .

31.	Leong C, Haszard JJ, Heath A-LM, Tannock GW, Lawley B, Cameron SL, Szymlek-Gay EA, Gray AR, Taylor BJ, Galland BC. Using compositional principal component analysis to describe children's gut microbiota in relation to diet and body composition. The American Journal of Clinical Nutrition. 2020;111:70-8. doi: 10.1093/ajcn/nqz270. PMID: 31711093.

32.	Bai J, Hu Y, Bruner DW. Composition of gut microbiota and its association with body mass index and lifestyle factors in a cohort of 7–18 years old children from the American Gut Project. Pediatric Obesity. 2019;14:e12480. doi: 10.1111/ijpo.12480. PMID: 30417607.

33.	Liang C, Guo M, Liu T, Zhou X, Gong P, Lyu L, Niu H, Wu Y, Chen S, Han X, Zhang L. Profiles of gut microbiota in children with obesity from Harbin, China and screening of strains with anti-obesity ability in vitro and in vivo. J Appl Microbiol. 2020;129:728-37. doi: 10.1111/jam.14639. PMID: 32162449.

34.	Mancera-Hurtado Y, Lopez-Contreras BE, Flores-Lopez R, Villamil-Ramirez H, del Rio-Navarro BE, Canizales-Quinteros S, Moran-Ramos S. A dysregulated bile acids pool is associated with metabolic syndrome and gut microbial dysbiosis in early adolescence. Obesity. 2023;31:2129-38. doi: [10.1002/oby.23797.](https://doi.org/10.1002/oby.23797.) PMID: 37403220.

34.	Kalliomäki M, Collado MC, Salminen S, Isolauri E. Early differences in fecal microbiota composition in children may predict overweight. Am J Clin Nutr. 2008;87:534-8. doi: 10.1093/ajcn/87.3.534. PMID: 18326589.

2. **Conference abstract(n=15)**

1.	Oguri K, Kasuga K, Yamaji S, Kitabayashi T, Sakai T, Nakano T. Comparison Of The Gut Microbiota Composition Between Obese And Non-obese Young Children Using 16s Gene Sequening: 2459 Board# 5 May 29 9: 30 AM-11: 30 AM. Medicine & Science in Sports & Exercise. 2020;52:665. doi: 10.1249/01.mss.0000682392.21258.94.

2.	Visuthranukul C, Sriswasdi S, Saengpanit P, Chomtho S. The composition of gut mcrobiota and its associations with BMI, adiposity and stool short chain fatty acids in obese children. Journal of Pediatric Gastroenterology and Nutrition. 2019;68:1237-8. doi: 10.1097/MPG.0000000000002403.

3.	Seong-Wong M, Abd Majid NH, Muhammad NA, Mustaffa N, Lee YY, Liong MT, Jayaraman T, Khine WWT, Lee YK. Diet and Obesity Rather than Ethnicity are The Determinants of Gut Microbiota Composition of Primary School Children in Kota Bharu, Malaysia. J Gastroenterol Hepatol: WILEY 111 RIVER ST, HOBOKEN 07030-5774, NJ USA, 2018:219-. doi: 10.1016/s0016-5085(19)38627-5.

4.	Youssef S, Singhal V, Stanford FC, Bredella M, Misra M. Differences in the Gut Microbiome Across a Range of Glucose Tolerance in Adolescents with Obesity. Horm Res Paediatr: KARGER ALLSCHWILERSTRASSE 10, CH-4009 BASEL, SWITZERLAND, 2020:13-.

5.	Rychkova L, Novikova E, Belkova N, Pogodina A, Romanitsa A, Feranchuk S. Features of gut microbiota composition in obese adolescents. Arch Dis Child. 2019;104:A11. doi: 10.1136/archdischild-2019-epa.25.

6.	Sotos M, Nadal I, Marti A, Martínez A, Martin-Matillas M, Campoy C, Puertollano M, Wärnberg J, Marcos A, Sanz Y. Gut microbes and obesity in adolescents. Proceedings of the Nutrition Society. 2008;67:E20. doi: 10.1126/science.368.6488.279-c.

7.	Michail S, Kenche H, Homer DC, Reo N. Gut microbial profile in pediatric non alcoholic fatty liver disease. Gastroenterology. 2011;5:S-118. doi: 10.1016/s0016-5085(11)60477-0.

8.	with Ai, Postdoctoral Research Fellow CUMC. The gut microbiome and childhood obesity: connecting the dots. Child Obes. 2015;11:227-30. doi: 10.1089/chi.2015.29000.nm. PMID: 26034811.

9.	Carrizales-Sánchez AK, García-Cayuela T, Hernández-Brenes C, Senés-Guerrero C. Gut microbiota associations with metabolic syndrome and relevance of its study in pediatric subjects. Gut Microbes. 2021;13:1960135. doi: 10.1080/19490976.2021.1960135. PMID: 34491882.

10.	Huddleston K, DeBoer M, Wong W, Gaiazov S, Fulchiero R, Saadoon R, Zhu W, Clemency N, Provenzana M, Vilboux T, Hourigan S. Identification of microbiome and clinical influences related to very early childhood obesity. Journal of Pediatric Gastroenterology and Nutrition. 2018;67:S225-S7. doi: 10.1097/MPG.0000000000002164.

11.	Ismail N, Mansour N, Ragab S, El-Baky A, Hamed M, Ayoub D. Intestinal microbiota and serum LPS level and correlation to fatty liver in obese Egyptian children. 2014.

12.	Touil A, Mehenni F, Leke A, Meddah B. Investigation into Changes in Gut Microbiota of Obesity in Children. OBESITY SURGERY: SPRINGER 233 SPRING ST, NEW YORK, NY 10013 USA, 2013:1180-.

13.	Abali ZY, Saygili S, Ozden T, Cantez S, Bas F, Ertekin V, Bundak R, Durmaz O, Darendeliler F. Nonalcoholic fatty liver disease and intestinal inflammation in obese children. Horm Res Paediatr. 2015;84:221. doi: 10.1159/000437032.

14.	Vankerckhoven V, Bervoets L, Van Hoorenbeeck K, Lammens C, Chapelle S, Vael C, Desager K, Goossens H. Relationship between the gut microbiota and obesity in children and adolescents. Clin Microbiol Infect. 2011;17:S91-S2. doi: 10.1111/j.1469-0691.2011.03557.x.

15.	Moran-Ramos S, Mancera-Hurtado Y, MacIas-Kauffer LK, Lopez-Contreas B, Villamil-Ramírez H, Ibarra-Gonzalez I, Vela-Amieva M, Aguilar-Salinas C, Canizales-Quinteros S. Gut microbiome and metabolic health in school age children: Perspectives for dietary interventions. Lifestyle Genomics. 2023;16:6-7. doi: 10.1159/000527546.

3. **Inaccessible(n=1)**

1.	Chen R, Ai Z, Yang X, Zhang Y, Yuan X. Altered gut microbiota in Obese children: sex associated signature. Horm Res Paediatr: KARGER ALLSCHWILERSTRASSE 10, CH-4009 BASEL, SWITZERLAND, 2019:158-9.

4. **Review(n=25)**

1.	Thomas-Eapen N. Childhood Obesity. Prim Care. 2021;48:505-15. doi: 10.1016/j.pop.2021.04.002. PMID: 34311854.

2.	Sanchez M, Panahi S, Tremblay A. Childhood obesity: a role for gut microbiota? International journal of environmental research and public health. 2015;12:162-75. doi: 10.3390/ijerph120100162. PMID: 25546278.

3.	Taylor MW. Devil in the detail: a closer look at childhood obesity and the gut microbiota. Environmental Microbiology. 2016;19:11-2. doi: 10.1111/1462-2920.13540. PMID: 27668702.

4.	Dreyer JL, Liebl AL. Early colonization of the gut microbiome and its relationship with obesity. Human Microbiome Journal. 2018;10:1-5. doi: 10.1016/j.humic.2018.08.002.

5.	Jian C, Carpén N, Helve O, de Vos WM, Korpela K, Salonen A. Early-life gut microbiota and its connection to metabolic health in children: Perspective on ecological drivers and need for quantitative approach. EBioMedicine. 2021;69:103475. doi: 10.1016/j.ebiom.2021.103475. PMID: 34256346.

6.	Feng-xiu O, Xu W. Factors influencing gut microbiota in early life and childhood obesity. JOURNAL OF SHANGHAI JIAOTONG UNIVERSITY (MEDICAL SCIENCE). 2016;36:1378. doi:10.3969/j.issn.1674-8115.2016.09.023.

7.	Kumbhare SV, Patangia DV, Patil RH, Shouche YS, Patil NP. Factors influencing the gut microbiome in children: from infancy to childhood. Journal of biosciences. 2019;44:1-19. doi: 10.1007/s12038-019-9860-z. PMID: 31180062.

8.	Nikolaeva I, Tsaregorodtsev A, Shaikhieva G. Formation of the intestinal microbiota of children and the factors that influence this process. Rossiyskiy Vestnik Perinatologii i Pediatrii (Russian Bulletin of Perinatology and Pediatrics). 2018;63:13-8. doi: 10.21508/1027-4065-2018-63-3-13-18.

9.	Murugesan S, Nirmalkar K, Hoyo-Vadillo C, García-Espitia M, Ramírez-Sánchez D, García-Mena J. Gut microbiome production of short-chain fatty acids and obesity in children. Eur J Clin Microbiol Infect Dis. 2018;37:621-5. doi: 10.1007/s10096-017-3143-0. PMID: 29196878.

10.	Yang Y-J, Ni Y-H. Gut microbiota and pediatric obesity/non-alcoholic fatty liver disease. J Formos Med Assoc. 2019;118:S55-S61. doi: 10.1016/j.jfma.2018.11.006. PMID: 30509561.

11.	Fonvig CE, Pihl AF, Hansen T, Pedersen OB, Holm J-C. Gut microbiota may influence childhood and adolescent onset obesity. Ugeskrift for Laeger. 2014;176. PMID: 25293572.

12.	Zamudio-Vázquez V, Ramírez-Mayans J, Toro-Monjaraz E, Cervantes-Bustamante R, Zarate-Mondragon F, Montijo-Barrios E, Cadena-León J, Cázares-Méndez J. Importance of gastrointestinal microbiota in children. Acta Pediátrica de México. 2017;38:49-62.

13.	Castañeda Guillot C. Intestinal microbiota and child health. Revista Cubana de Pediatria. 2018;90:94-110.

14.	Buccigrossi V, Nicastro E, Guarino A. Intestinal microbiota composition in children. Probiotic Bacteria and Their Effect on Human Health and Well-Being: Karger Publishers, 2013:9-16.

15.	Kaneko K. Intestinal Microbiota of Childhood: Dysbiosis and Diseases. Journal of Pediatric Biochemistry. 2015;5:039-40. doi: 10.1055/s-0035-1564572.

16.	Luoto R, Collado MC, Laitinen K, Salminen S, Isolauri E. Metabolic Syndrome and Obesity in Children. In: Guarino A, Quigley EMM, Walker WA, eds. Probiotic Bacteria and Their Effect on Human Health and Well-Being. Basel: Karger, 2013:95-102. doi: 10.1159/000345738

17.	Ziganshina AA, Rylova NV. Microbiota balance in child’s intestines - A key to good health. Pediatriya - Zhurnal im GN Speranskogo. 2019;98:134-9. doi: 10.24110/0031-403X-2019-98-6-134-139.

18.	Videhult FK, West CE. Nutrition, gut microbiota and child health outcomes. Curr Opin Clin Nutr Metab Care. 2016;19:208-13. doi: 10.1097/mco.0000000000000266. PMID: 26870888.

19.	Phelan S, Ventura AK. Prenatal risk factors for childhood obesity. Childhood Obesity: Causes, Consequences, and Intervention Approaches, 2016:127-35.

20.	Durmaz B. Relationship between obesity and gut microbiota. Türk Hijyen ve Deneysel Biyoloji Dergisi. 2019;76:353-60. doi: 10.2174/0929867327666200721160313. PMID: 32693755.

21.	Wang W, Yan Y, Yu F, Zhang W, Su S. Role of oral and gut microbiota in childhood obesity. Folia Microbiol (Praha). 2023;68:197-206. doi: 10.1007/s12223-023-01033-3. PMID: 36626083.

22.	Pihl AF, Fonvig CE, Stjernholm T, Hansen T, Pedersen O, Holm JC. The Role of the Gut Microbiota in Childhood Obesity. Child Obes. 2016;12:292-9. doi: 10.1089/chi.2015.0220. PMID: 27058515.

23.	Tokuhara D. Role of the Gut Microbiota in Regulating Non-alcoholic Fatty Liver Disease in Children and Adolescents. Front Nutr. 2021;8:700058. doi: 10.3389/fnut.2021.700058. PMID: 34250000.

24.	Zhang S, Dang Y. Roles of gut microbiota and metabolites in overweight and obesity of children. Front Endocrinol (Lausanne). 2022;13:994930. doi: 10.3389/fendo.2022.994930. PMID: 36157438.

25.	Dinsmoor AM, Aguilar-Lopez M, Khan NA, Donovan SM. A systematic review of dietary influences on fecal microbiota composition and function among healthy humans 1–20 years of age. Adv Nutr. 2021;12:1734-50. doi: 10.1093/advances/nmab047. PMID: 33951139

**Supplemental Figure 1: Reported gut microbiota characteristics in children with obesity (the number of articles on the same gut microbiota study ≥ 3 was counted).** Orange, green and grey indicate a significant increase, significant decrease, or no significant difference in the relative abundance of gut microbiota in children with obesity compared to the normal group, respectively. The “N” indicates the sample size. The “n” indicates the number of included studies.

**Supplemental Figure 2:** **Assessment of publication bias in α-diversity meta-analyses.**

**Supplemental Figure 3: Comparative analysis of α-diversity forest plots between obese children and control subjects.**

**Supplemental Figure 4: Sensitivity analysis of α-diversity.**

**Supplemental Figure 5: A α-diversity forest plot comparing obese children with control groups based on region, age, and BMI subgroups.**

**Supplemental Figure 6: Comparative analysis of forest plots at the phyla level between obese children and control subjects.**

**Supplemental Figure 7: Sensitivity analysis at the phyla level.**

**Supplemental Figure 8: The assessment of publication bias for meta-analyses at the Phyla level.**

**Supplemental Figure 9: A Phyla level forest plot comparing obese children with control groups based on region, age, and BMI subgroups.**

**Supplemental Figure 10:** **Comparative forest plots at the genera level between obese children and control subjects.**

**Supplemental Figure 11:** **Sensitivity analysis at the level of genera.**

**Supplemental Figure 12:** **A genera level forest plot comparing obese children with control groups based on region, age, and BMI subgroups.**

**Supplemental Figure 13: Comparative analysis of forest plots at the species level between obese children and control subjects.**

**Supplemental Figure 14: Sensitivity analysis at the species level.**

**Supplemental Figure 15:** **A species level forest plot comparing obese children with control groups based on region, age, and BMI subgroups.**
